# Comparative safety of different recommended doses of sodium–glucose cotransporter 2 inhibitors in patients with type 2 diabetes mellitus: a systematic review and network meta-analysis of randomized clinical trials

**DOI:** 10.3389/fendo.2023.1256548

**Published:** 2023-11-10

**Authors:** Lu Chen, Qingxia Xue, Chunyan Yan, Bingying Tang, Lu Wang, Bei Zhang, Quan Zhao

**Affiliations:** Department of Pharmacy, Yantai Yuhuangding Hospital, Shandong, China

**Keywords:** sodium-glucose cotransporter 2 inhibitors, type 2 diabetes mellitus, adverse events, randomized controlled trials, network meta-analysis

## Abstract

**Objective:**

The safety results of different recommended doses of sodium-glucose cotransporter 2 inhibitors (SGLT-2i) for patients with type 2 diabetes mellitus (T2DM) remain uncertain. This study aims to comprehensively estimate and rank the relative safety outcomes with different doses of SGLT-2i for T2DM.

**Methods:**

PubMed, Embase, the Cochrane Central Register of Controlled Trials, ClinicalTrials.gov, Chinese National Knowledge Infrastructure, WanFang database, and SinoMed database were searched from the inception to 31 May 2023. We included double-blind randomized controlled trials (RCTs) comparing SGLT-2i with placebo or another antihyperglycemic as oral monotherapy in the adults with a diagnosis of T2DM.

**Results:**

Twenty-five RCTs with 12,990 patients randomly assigned to 10 pharmacological interventions and placebo were included. Regarding genital infections (GI), all SGLT-2i, except for ertugliflozin and ipragliflozin, were associated with a higher risk of GI compared to placebo. Empagliflozin 10mg/d (88.2%, odds ratio [OR] 7.90, 95% credible interval [CrI] 3.39 to 22.08) may be the riskiest, followed by empagliflozin 25mg/d (83.4%, OR 7.22, 95%CrI 3.11 to 20.04)) and canagliflozin 300mg/d (70.8%, OR 5.33, 95%CrI 2.25 to 13.83) based on probability rankings. Additionally, dapagliflozin 10mg/d ranked highest for urinary tract infections (UTI, OR 2.11, 95%CrI 1.20 to 3.79, 87.2%), renal impairment (80.7%), and nasopharyngitis (81.6%) when compared to placebo and other treatments. No increased risk of harm was observed with different doses of SGLT-2i regarding hypoglycemia, acute kidney injury, diabetic ketoacidosis, or fracture. Further subgroup analysis by gender revealed no significantly increased risk of UTI. Dapagliflozin 10mg/d (91.9%) and canagliflozin 300mg/d (88.8%) ranked first in the female and male subgroups, respectively, according to the probability rankings for GI.

**Conclusion:**

Current evidence indicated that SGLT-2i did not significantly increase the risk of harm when comparing different doses, except for dapagliflozin 10mg/d, which showed an increased risk of UTI and may be associated with a higher risk of renal impairment and nasopharyngitis. Additionally, compared with placebo and metformin, the risk of GI was notably elevated for empagliflozin 10mg/d, canagliflozin 300mg/d, and dapagliflozin 10mg/d. However, it is important to note that further well-designed RCTs with larger sample sizes are necessary to verify and optimize the current body of evidence.

**Systematic Review Registration:**

https://www.crd.york.ac.uk/PROSPERO/, identifier CRD42023396023.

## 1 Introduction

Type 2 diabetes mellitus (T2DM) is a chronic disease that results from a combination of insulin resistance and insulin deficiency caused by progressive beta-cell failure. It is associated with both microvascular and macrovascular complications, causing significant psychological and physical distress for patients and carers while placing a substantial burden on healthcare systems (1). According to the International Diabetes Federation, in 2021, 537 million adults were estimated with diabetes worldwide, with China accounting for approximately 26% of this total, equating to 141 million adults, of which T2DM represents 90% of all diabetes cases in China (2, 3).

The National Institute for Health and Care Excellence (NICE) clinical practice guidelines recommend metformin, along with lifestyle modifications, as the first-line treatment for T2DM (4). However, some patients may be intolerant to metformin due to gastrointestinal events, and metformin alone may not be sufficient to achieve or maintain glycemic goals (5). Based on increasingly high-quality randomized clinical trials (RCTs) and meta-analyses, sodium-glucose transporter 2 inhibitors (SGLT-2i) were recommended as one of the effective hypoglycemic agents for second-line therapy after metformin failure or intolerance (6). SGLT-2i reduce hyperglycemia in T2DM patients by inhibiting renal glucose reabsorption and increasing glucose excretion in the urine. Currently, SGLT-2i are strongly recommended as the preferred initial medical treatment in combination with metformin for T2DM patients, as they have demonstrated favorable effects on blood glucose control, cardiovascular outcomes, and renal benefits. These effects have led to reduce the 3-point major adverse cardiovascular effect (MACE), total mortality, and heart failure, as highlighted by clinical practice guidelines (7, 8). However, post-marketing adverse event reports have raised concerns about the safety of SGLT-2i, including adverse effects such as genital and urinary tract infections, amputation, and diabetic ketoacidosis (DKA). Regulatory authorities have issued drug safety communications regarding the potential risk of acute kidney injury (AKI), DKA, hypoglycemia, bone fractures, and Fournier’s gangrene associated with the use of SGLT-2i (9, 10). Although several systematic reviews and meta-analyses have been conducted to assess the safety outcomes of SGLT-2i, the findings have not proved consistent across trials, and most studies have primarily focused on comparing the class of SGLT-2i (11–13). To date, there is a lack of comprehensive studies that have analyzed the risk of adverse outcomes related to all approved SGLT-2i, particularly in relation to the different recommended doses of each SGLT-2i. As a result, clinicians and patients are left uncertain about the potential health outcomes. Therefore, it is crucial for clinicians and policymakers to continuously integrate new pharmacotherapeutic evidence to optimize health outcomes.

The aim of this study is to conduct a Bayesian network meta-analysis (NMA) to estimate and rank the relative safety outcomes associated with different recommended doses of each approved SGLT-2i for T2DM. The results of this analysis are expected to provide valuable insights for clinical decision-making, enabling the development of optimal treatment strategies for patients with T2DM in the future.

## 2 Methods

The NMA was performed in accordance with Preferred Reporting Items for Systematic Review and Meta-Analysis extension statement for NMA (**Table S1**) (14). This study was registered in PROSPERO (CRD42023396023).

### 2.1 Search strategy and selection criteria

PubMed, Embase, the Cochrane Central Register of Controlled Trials, ClinicalTrials.gov, China National Knowledge Infrastructure, WanFang database, and SinoMed database were searched from inception to 31 May 2023, using the term “SGLT-2”, “T2DM”, “randomized controlled trials”, and their synonyms shown in **Supplementary Material** (**Table S2**). Additionally, a manual search of reference lists of relevant studies was performed to identify further eligible studies. We only identified double-blind RCTs comparing any marketable SGLT-2i with either placebo or another active antihyperglycemic as oral monotherapy in the adults (≥18 years old) with a diagnosis of T2DM, and the languages searched were limited to either English or Chinese. To be eligible, one of the RCT study groups needed to receive one of the recommended doses of SGLT-2i according to the drug instructions and Food and Drug Administration (FDA) guidelines. These recommended doses included canagliflozin (100mg/d, 300mg/d), dapagliflozin (5mg/d, 10mg/d), empagliflozin (10 mg/d, 25 mg/d), ertugliflozin (5 mg/d, 15mg/d), ipragliflozin (50mg/d, 100mg/d), luseogliflozin (2.5mg/d, 5mg/d), tofogliflozin (20 mg/d), or henagliflozin (5mg/d, 10mg/d).

We excluded trials that patients with severe hepatic impairment (Child-Pugh class C), severe renal dysfunction or end-stage renal failure (estimated glomerular filtration rate/eGFR < 30mL/min/1.73 m^2^), pregnancy or lactation, insulin therapy, or allergies or contraindication to the study drugs. Additionally, trials that combined treatment with other antihyperglycemic drugs during the study period were also excluded.

The outcomes of this study included adverse events (AEs) of special interest, such as confirmed hypoglycemic events (plasma glucose ≤ 3.9 mmol/l and/or requiring assistance), as well as AEs related to urinary tract infections (UTI), genital infections (GI), renal-related AEs, bone fractures, amputations, DKA, and nasopharyngitis.

Four reviewers (C.L., X.Q.X. Y.C.Y., and W.L.) independently screened the reports against pre-designed eligibility criteria, and any disagreements were resolved through discussion, consulting another reviewer (Z.B. or Z.Q.).

### 2.2 Data extraction

Three reviewers (C.L., X.Q.X., T.B.Y.) independently screened each trial by reviewing titles, abstracts, and full text using standardized and piloted forms. The baseline information was extracted, including the first author, the publication year, the clinical trials number, participants characteristics (including age, gender, BMI, eGFR, and sample size), intervention and comparison groups with dosage and usage, as well as the outcomes. Discrepancies were resolved through discussion or by a third reviewer (Z.B. or Z.Q.).

### 2.3 Risk-of-Bias assessments

Two reviewers (C.L. and X.Q.X.) independently assessed the risk of bias using the Cochrane Collaboration’s Risk of Bias 2 (RoB V.2.0) tool, encompassing domains such as randomization process, deviations from intended interventions, missing outcome data, outcome measurement, and selection of the reported result (15). Each study was classified as low risk, some concerns, or high risk. Discrepancies were resolved through discussion or by a third reviewer (Z.B. or Z.Q.).

### 2.4 Statistical analysis

We estimated intervention effects by calculating odds ratios (OR) with 95% credible interval (CrI). We performed NMA using Bayesian random effect models with the Markov chain Monte Carlo simulation method for interventions that connected to an evidence network by data available from ≥ 2 studies. For outcomes with inadequate network structure, pairwise meta-analyses were conducted instead. Heterogeneity among studies was assessed using the Q test and I² statistic, with significance defined as p < 0.10 and I² ≥ 50% (16). Consistency between direct and indirect evidence in the existing closed loops was examined using the node-splitting approach. A meta-regression method was employed to analyze differences in baseline characteristics when at least 10 studies included.

Model convergence was evaluated via visual inspection of four chains, considering the Brooks-Gelman-Rubin diagnostic, as well as trace and density plots (17). Within the Bayesian framework, all interventions were ranked using the surface under the cumulative ranking (SUCRA) curve (18). A larger SUCRA value, the higher the risk of such drugs occurring in this outcome.

Subgroup analyses were performed by gender when sufficient information was available. Additionally, Sensitivity analysis was performed by excluding high risk of bias of studies. A comparison-adjusted funnel plot and Egger test were used to evaluate small-study effects for individual outcomes when at least 10 eligible studies were available (19). Statistical significance was set at p < 0.05. All NMAs were performed using OpenBUGS version 3.2.3 and the Stata software version 15.0.

## 3 Results

### 3.1 Study selection and characteristics

**Figure 1:**
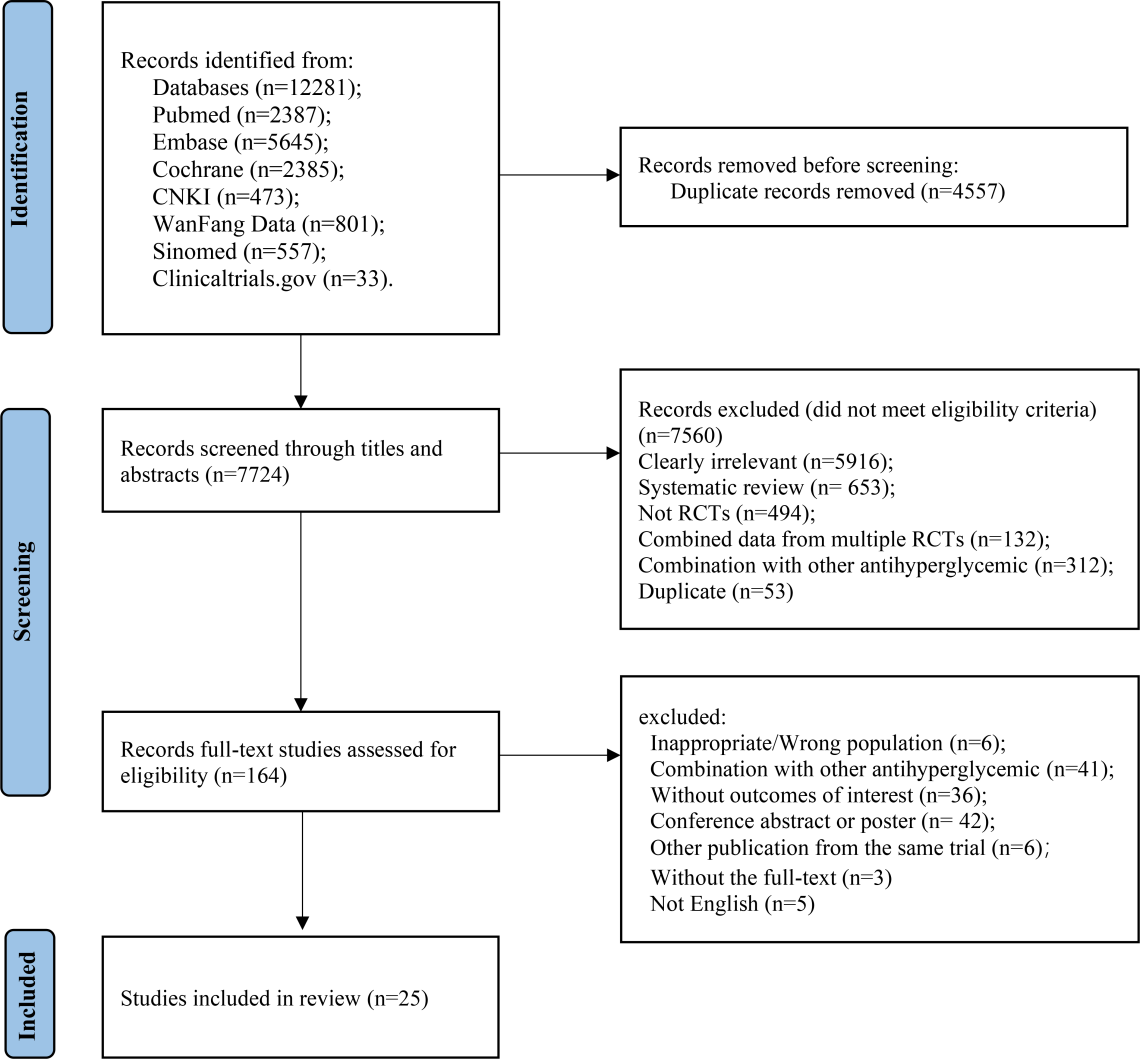
Study selection process.

**Figure 2:**
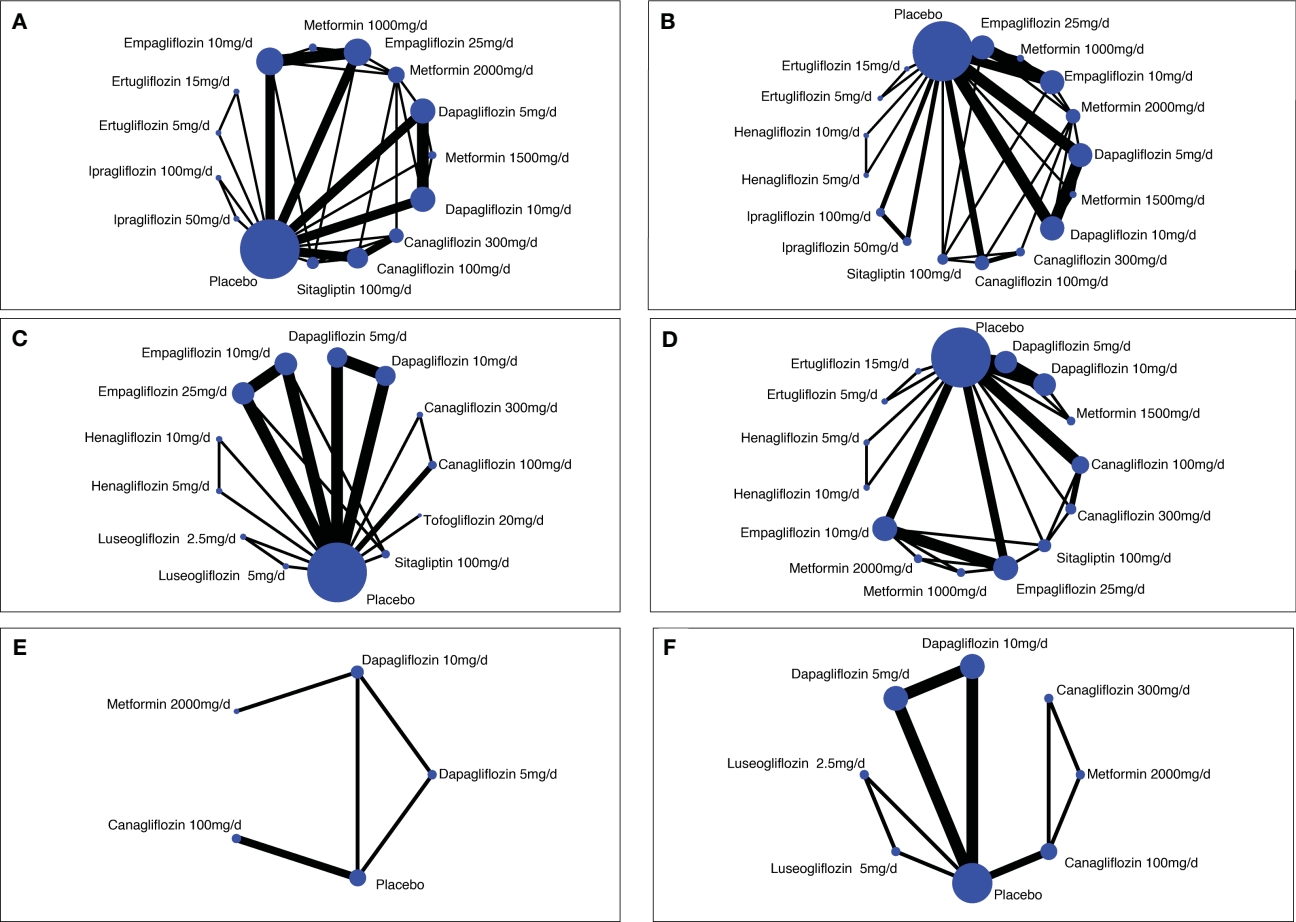
Network diagrams of comparisons on different safety outcomes. **(A)** Genital infections; **(B)** unitary tract infections; **(C)** nasopharyngitis; **(D)** hypoglycemia; **(E)** bone fracture; **(F)** renal-related adverse events. Each circular node represents a type of treatment. The node size corresponds to the number of patients for each treatment. Lines indicate direct head-to-head comparisons, and the line width corresponds to the number of trials in the comparison.

A total of 7,486 studies were identified after removing duplications, 164 potentially eligible studies underwent full-text review. Following the application of eligibility criteria, 25 RCTs involving 12,990 patients were included in NMA. The study selection process is illustrated in **Figure 1**. A network of eligible comparisons for the multiple treatment meta-analysis of each safety outcome was constructed (**Figure 2**). The included RCTs compared 11 treatments, including canagliflozin, dapagliflozin, empagliflozin, ertugliflozin, ipragliflozin, luseogliflozin, tofogliflozin, henagliflozin, metformin, sitagliptin, and placebo. The baseline characteristics of the included studies are summarized in **Table 1**. Notably, the interested outcomes of two RCTs (NCT00643851 and NCT00859898) were extracted in one study (32). Among these, three studies were four-arm trials, 15 were three-arm trials, and the remainder were double-arm trials. Specifically, five RCTs compared dapagliflozin to placebo, five compared canagliflozin to placebo, three compared empagliflozin to placebo, one compared ertugliflozin to placebo, two compared ipragliflozin to placebo, one compared luseogliflozin to placebo, one compared tofogliflozin to placebo, one compared henagliflozin to placebo, and four respectively compared dapagliflozin, empagliflozin, and canagliflozin to metformin. The mean sample size of the included studies was 519 patients, ranging from 22 to 4,307 patients, and the mean age was 55.6 years (standard deviation: 3.6). The duration of trials ranged from 2 to 287 weeks (median: 26 weeks).

**Table 1:** Study characteristics of the included trials and participants.

First author (year)	Clinical trial number	Sample size (T/C)	Age (mean ± SD)	Gender (T/C, Female%)	BMI (T/C, kg/m^2^)	HbA1c/%	eGFR/ m L·min^-1^· (1.73 m^2^) ^-1^	Treatment/Control (dosage)	Duration of trial (weeks)	Outcomes
Stenlöf 2013 (20)	NCT01081834	195 197 192	55.1 ± 10.8 55.3 ± 10.2 55.7 ± 10.9	58.5% 54.8% 54.2%	31.3 ± 6.6 31.7 ± 6.0 31.8 ± 6.0	8.1 ± 1.0 8.0 ± 1.0 8.0 ± 1.0	88.5 ± 20.2 86.6 ± 19.1 86.0 ± 21.5	Canagliflozin (100 mg/d, 300mg/d); Sitagliptin 100 mg/d	52	UTI, GI, hypoglycemia
Rosenstock 2016 (21)	NCT01809327	237 238 237	54.0 ± 10.7 55.8 ± 9.6 55.2 ± 9.8	55.7% 47.5% 51.1%	32.4 ± 5.4 32.6 ± 5.8 33.0 ± 6.0	8.8 ± 1.2 8.8 ± 1.2 8.8 ± 1.2	90.0 ± 19.0 85.0 ± 18.0 87.0 ± 19.0	Canagliflozin (100mg/d, 300mg/d); Metformin 2000mg/d	30	UTI, GI, hypoglycemia, and Renal-related AEs
Inagaki 2013 (22)	NCT01022112	74 75 75	57.7 ± 10.5 57.1 ± 10.1 57.7 ± 11.0	29.7% 26.7% 28.0%	25.6 ± 4.6 25.9 ± 3.7 26.4 ± 4.3	8.0 ± 0.9 8.2 ± 0.8 8.0 ± 0.8	86.9 ± 15.5 86.9 ± 15.2 83.0 ± 16.5	Canagliflozin (100mg/d, 300mg/d); Placebo	14	GI, nasopharyngitis, hypoglycemia
Wada 2022 (23)	NCT03436693	154 154	62.5 ± 10.5 62.4 ± 11.1	25.3% 16.2%	26.7 ± 4.4 27.1 ± 4.5	7.7 ± 1.1 7.8 ± 1.0	56.3 ± 15.5 55.2 ± 13.6	Canagliflozin 100mg/d; Placebo	108	UTI, GI, fracture, hypoglycemia, DKA, renal-related AEs
Iijima 2015 (24)	NCT00707954	12 10	52.1 ± 7.6 57.6 ± 6.3	0% 20%	25.2 ± 2.4 25.7 ± 3.4	8.3 ± 0.8 8.9 ± 1.2	NR	Canagliflozin 100mg/d; Placebo	2	Nasopharyngitis
Perkovic 2019 (25)	NCT02065791	2200 2197	62.9 ± 9.2 63.2 ± 9.2	34.6% 33.3%	31.4 ± 6.2 31.3 ± 6.2	8.3 ± 1.3 8.3 ± 1.3	56.3 ± 18.2 56.0 ± 18.3	Canagliflozin 100mg/d; Placebo	287	UTI, GI, hypoglycemia, fracture, amputation, acute kidney injury, DKA
Inagaki 2014 (26)	NCT01413204	90 93	58.4 ± 10.4 58.2 ± 11.0	34.4% 35.5%	25.6 ± 4.2 25.8 ± 4.4	8.0 ± 0.7 8.0 ± 0.7	81.4 ± 13.8 84.7 ± 13.7	Canagliflozin 100mg/d; Placebo	26	UTI, GI, hypoglycemia
Ji 2014 (27)	NCT01095653	128 133 132	53.0 ± 11.1 51.2 ± 9.89 49.9 ± 10.9	34.4% 35.3% 34.1%	25.2± 3.3 25.8 ± 3.4 25.9 ± 3.6	8.1 ± 0.7 8.3 ± 0.9 8.3 ± 0.9	91.6 ± 17.1 91.7 ± 20.2 94.1 ± 17.7	Dapagliflozin (5 mg/d, 10 mg/d); Placebo	28	UTI, GI, hypoglycemia, renal impairment, nasopharyngitis
Kaku 2013 (28)	NCT00972244	58 52 54	58.0 ± 9.5 56.5 ± 11.5 58.4 ± 10.0	19.0% 25.0% 20.4%	NR	8.0 ± 0.7 8.2 ± 0.7 8.1 ± 0.7	NR	Dapagliflozin (5 mg/d, 10 mg/d); Placebo	16	UTI, GI, hypoglycemia, nasopharyngitis
List 2009 (29)	NCT00263276	58 47 54 56	55.0 ± 12.0 54.0 ± 9.0 53.0 ± 11.0 54.0 ± 9.0	52.0% 47.0% 44.0% 52.0%	32.0 ± 5.0 31.0 ± 5.0 32.0 ± 5.0 32.0 ± 5.0	8.0 ± 0.9 8.0 ± 0.8 7.9 ± 0.9 7.6 ± 0.8	>60	Dapagliflozin (5 mg/d, 10mg/d); Placebo Metformin 1500 mg/d	16	UTI, GI, hypoglycemia
Kaku 2014 (30)	NA	86 88 87	58.6 ± 10.4 57.5 ± 9.3 60.4 ± 9.7	41.9% 39.8% 40.2%	24.9 ± 3.9 26.1 ± 4.5 25.2 ± 4.4	7.5 ± 0.7 7.5 ± 0.6 7.5 ± 0.6	66.5 ± 11.4 66.9 ± 11.0 67.8 ± 12.4	Dapagliflozin (5mg/d, 10mg/d); Placebo	27	UTI, GI, renal impairment, nasopharyngitis, rib fracture
Bailey 2015 (31)	NCT00528372	64 70 75	52.6 ± 10.9 50.6 ± 10.0 52.7 ± 10.3	51.6% 51.4% 58.7%	NR	7.9 ± 0.9 8.0 ± 1.0 7.8 ± 0.9	NR	Dapagliflozin (5mg/d,10mg/d); Placebo	102	UTI, GI, nasopharyngitis, hypoglycemia, renal impairment/failure
Henry 2012 (32)	NCT00859898	219 208	51.1 ± 11.53 52.7 ± 10.38	52.1% 53.4%	NA	9.0 ± 1.3 9.0 ± 1.3	NA	Dapagliflozin 10 mg/d; Metformin 2000 mg/d	28	GI, UTI, hypoglycemia, renal impairment or failure, fractures
Henry 2012 (32)	NCT00643851	203 201	52.3 ± 10.20 51.8 ± 9.80	54.7% 52.7%	NA	9.1 ± 1.4 9.2 ± 1.3	NA	Dapagliflozin 5 mg/d Metformin 2000 mg/d	28	GI, UTI
Hadjadj 2016 (33)	NCT01719003	172 167 171 170	53.1 ± 10.7 53.3 ± 10.7 53.4 ± 10.9 51.6 ± 10.8	42.6% 49.4% 48.8% 43.9%	30.3 ± 5.2 30.6 ± 5.9 30.3 ± 5.8 30.5 ± 5.9	8.6 ± 1.2 8.9 ± 1.3 8.7± 1.0 8.6 ± 1.1	94.0 ± 21.5 91.7 ± 19.5 90.9 ± 19.4 93.2 ± 20.2	Empagliflozin (10mg/d, 25mg/d); Metformin (1000mg/d, 2000mg/d)	25	GI, UTI, hypoglycemia
Tikkanen 2015 (34)	NCT01370005	276 276 271	60.6 ± 8.5 59.9 ± 9.7 60.3 ± 8.8	38.0% 43.5% 38.0%	32.4 ± 5.3 33.0 ± 5.0 32.4 ± 4.9	7.9 ± 0.8 7.9 ± 0.7 7.9 ± 0.7	83.4 ± 16.7 83.5 ± 17.8 85.0 ± 17.0	Empagliflozin (10mg/d, 25mg/d); Placebo	14	UTI, GI, nasopharyngitis, hypoglycemia
Kadowaki 2014 (35)	NCT01193218	109 109 109	57.9 ± 9.4 57.2 ± 9.7 58.7 ± 8.7	29.4% 22.9% 26.6%	25.3 ± 4.4 25.1 ± 3.8 25.6 ± 3.4	7.9 ± 0.7 7.9 ± 0.8 7.9 ± 0.7	85.8 ± 14.6 85.2 ± 15.8 84.6 ± 14.9	Empagliflozin (10mg/d, 25mg/d); Placebo	13	UTI, GI, nasopharyngitis, hypoglycemia
Ferrannini 2013 (36)	NCT00789035	81 82 82	58 (30–76)^*^ 57 (30–79)^*^ 58 (28–80)^*^	41% 41% 37%	28.1 (21.5–39.3) ^*^ 28.3 (20.1–38.8) ^*^ 28.8 (20.7–39.6) ^*^	8.0 ± 0.8 7.8 ± 0.8 7.8 ± 0.8	NR	Empagliflozin (10mg/d, 25mg/d); Placebo	13	UTI, GI, nasopharyngitis
Roden 2015 (37)	NCT01289990	224 224 223 228	56.2 ± 11.6 53.8 ± 11.6 55.1 ± 9.9 54.9 ± 10.9	36.6% 35.3% 36.8% 46.1%	28.3 ± 5.5 28.2 ± 5.5 28.2 ± 5.2 28.7 ± 6.2	7.9 ± 0.9 7.9 ± 0.8 7.8 ± 0.8 7.9 ± 0.8	87.7 ± 19.2 87.6 ± 18.3 87.6 ± 17.3 86.8 ± 17.9	Empagliflozin (10mg/d, 25mg/d); Sitagliptin 100 mg/d; Placebo	80	UTI, GI, nasopharyngitis, hypoglycemia
Terra 2017 (38)	NCT01958671	156 152 153	56.8 ± 11.4 56.2 ± 10.8 56.1 ± 10.9	42.9% 40.8% 46.4%	33.2 ± 7.4 32.5 ± 5.7 33.3 ± 6.8	8.16 ± 0.88 8.35 ± 1.12 8.11 ± 0.92	88.5 ± 18.4 88.3 ± 18.0 86.2 ± 19.4	Ertugliflozin (5mg/d,15mg/d); Placebo	26	UTI, GI, hypoglycemia
Lu 2021 (39)	NCT03159052	150 151 151	53.3 ± 9.6 52.2 ± 9.4 52.4 ± 10.2	41.3% 23.8% 33.8%	25.4 ± 3.1 25.5 ± 3.1 26.0 ± 2.9	8.6± 0.9 8.6 ± 0.9 8.6 ± 0.9	120.0 ± 32.0 122.0 ± 31.0 124.0 ± 30.0	Henagliflozin (5mg/d, 10mg/d); Placebo	28	UTI, hypoglycemia, nasopharyngitis
Kashiwagi 2014 (40)	NCT00621868	72 72 69	55.9 ± 11.4 56.0 ± 10.4 55.2 ± 9.7	40.3% 31.9% 29.0%	25.8 ± 3.5 25.9 ± 3.8 25.1 ± 3.4	8.3 ± 0.8 8.2 ± 0.8 8.4 ± 0.8	NR	Ipragliflozin (50mg/d, 100mg/d); Placebo	18	UTI, GI
Schwartz 2011 (41)	NR	12 12 13	57.7 ± 9.1 57.3 ± 9.5 53.3 ± 11.9	58.3% 41.7% 30.8%	30.6 ± 4.62 34.1 ± 5.28 32.4 ± 4.32	NR	95.7 ± 25.6 99.8 ± 22.7 105.7 ± 21.4	Ipragliflozin (50mg/d, 100mg/d); Placebo	4	UTI
Seino 2014 (42)	JapicCTI-090908	60 61 61 54	58.3 ± 9.4 56.8 ± 9.3 57.6 ± 11.0	42.6% 27.9% 25.9%	24.8 ± 3.6 24.5 ± 3.2 25.2 ± 4.3	8.1 ± 0.9 8.2 ± 1.0 7.9 ± 0.7	NR	Luseogliflozin (2.5 mg/d, 5 mg/d); Placebo	12	Nasopharyngitis, renal-related AEs
Kaku 2014 (43)	Japic CTI-101349	58 56	56.6 ± 10.2 56.8 ± 9.9	32.8% 33.9%	25.0 ± 4.5 26.0 ± 4.1	8.3 ± 0.8 8.4 ± 0.8	86.8 ± 19.6 83.8 ± 17.7	Tofogliflozin 20 mg/d Placebo	26	Nasopharyngitis

^*^Median (range); UTI, urinary tract infection; GI, genital infection; DKA, Diabetic ketoacidosis; AEs, adverse events; NR, not reported.

The results of the risk of bias assessment are presented in **Table S3** and **Figure S1**. Overall, 16 (64%) studies had a low risk of bias, and 9 (36%) studies were evaluated as having some concerns due to a lack of allocation concealment. All studies were at low risk of bias from blinding, selective outcome reporting, and missing outcome data.

### 3.2 Primary analysis

#### 3.2.1 Genital infections

Twenty RCTs involving 12,076 patients provided data on GI (20–23, 25–38, 40), the direct and network results were summarized in the **Supplementary Material Table S4** and **Table S5A**. Based on the synthesized results, all SGLT-2i, except for ertugliflozin 5mg/d and ipragliflozin 100mg/d, were associated with a higher risk of GI compared to placebo and metformin 2000mg/d, respectively. Patients taking canagliflozin (100mg/d, 300mg/d), empagliflozin (10mg/d, 25mg/d), dapagliflozin 10mg/d, and ipragliflozin 50mg/d had a higher risk of GI compared to those taking sitagliptin 100mg/d. No significant association was observed between different SGLT-2i, regardless of the recommended dose. Furthermore, the ranking of treatments based on SUCRA values (**Table S6**) showed that ipragliflozin 50mg/d (95.7%) ranked first among the SGLT-2i, followed by ipragliflozin 100mg/d (89.9%), empagliflozin 10mg/d (75.9%), empagliflozin 25mg/d (72.2%), and canagliflozin 300mg/d (60.7%). However, this pooled result may be influenced by only one trial each for ipragliflozin and ertugliflozin, both with small sample sizes, leading to limited precision. After excluding these two trials, the overall pooled estimate remained stable. The SUCRA values indicated that empagliflozin 10mg/d (88.2%, compared to placebo: OR 7.86, 95% CrI 3.45 to 21.03) ranked first, followed by empagliflozin 25mg/d (83.4%, compared to placebo: OR 7.23, 95% CrI 3.08 to 19.36), canagliflozin 300mg/d (70.8%, compared to placebo: OR 5.46, 95% CrI 2.30 to 13.62), and dapagliflozin 10mg/d (69.7%, compared to placebo: OR 5.09, 95% CrI 1.92 to 13.66). The node-splitting analysis revealed inconsistencies in three out of the 21 comparisons, and the comparison-adjusted funnel plot and Egger’s test (p=0.10) indicated no asymmetry (**Table S7A**; **Figure S3A**).

#### 3.2.2 Urinary tract infections

Twenty-one RCTs involving 12,399 patients reported UTI (20, 21, 23, 25–29, 31–41, 43). The direct comparison showed that only dapagliflozin 10mg/d was significantly associated with a higher risk of UTI compared to placebo (OR 2.14, 95% CrI 1.03 to 4.44) or metformin 2000mg/d (OR 2.72, 95% CrI 1.23 to 6.00) (**Table S4**). The synthesized results indicated that dapagliflozin 10mg/d was associated with a higher risk of UTI compared to empagliflozin 10mg/d (OR 2.40, 95% CrI 1.26 to 4.64), empagliflozin 25mg/d (OR 2.27, 95% CrI 1.20 to 4.36), canagliflozin 100mg/d (OR 1.90, 95% CrI 1.06 to 3.49), ertugliflozin 15mg/d (OR 4.97, 95% CrI 1.59 to 17.09), metformin 2000mg/d (OR 2.00, 95% CrI 1.18 to 3.44), sitagliptin 100mg/d (OR 2.44, 95% CrI 1.21 to 5.02), and placebo (OR 2.11, 95% CrI 1.20 to 3.79), whereas there was no significant association between other treatments (**Table S5B**). Moreover, based on the SUCRA values ranking of treatments (**Table S6**), dapagliflozin 10mg/d (87.2%) ranked first among the SGLT-2i, followed by ipragliflozin 50mg/d (80.6%) and dapagliflozin 5mg/d (71.1%). The node-splitting method revealed no inconsistencies between direct and indirect evidence, and the comparison-adjusted funnel plot and Egger’s test (p=0.67) indicated no asymmetry (**Table S7B**; **Figure S3B**).

#### 3.2.3 Hypoglycemia

Seventeen RCTs involving 11,464 patients reported hypoglycemia (20–23, 25–29, 31–35, 37–39). The direct comparison did not show any treatments significantly associated with a higher risk of hypoglycemia compared to placebo or other active treatments (**Table S4**). The synthesized results indicated that all SGLT-2s, except for canagliflozin 100mg/d (OR 3.12, 95%CrI 1.18 to 8.36), were not significantly associated with a higher risk of hypoglycemia compared to placebo (**Table S5C**). It is worth noting that other drugs, such as metformin 1000mg/d, showed a lower risk of hypoglycemia compared to placebo and other active drugs. However, due to the low number of patients included, the statistical certainty was low, resulting in a wide credible interval. Based on the SUCRA values ranking of treatments, metformin 2000mg/d (82.7%) ranked first, followed by canagliflozin 100mg/d (80.6%), and canagliflozin 300mg/d (72.1%) (**Table S6**). The node-splitting analysis revealed inconsistencies in one of the 15 comparisons, and the comparison-adjusted funnel plot and Egger’s test (p=0.37) indicated no asymmetry (**Table S7C**; **Figure S3C**).

#### 3.2.4 Nasopharyngitis

Thirteen RCTs involving 4,315 patients reported nasopharyngitis (22, 24, 27, 28, 31, 34–37, 39, 42–44). Both the direct comparison and the synthesized results from the NMA did not show any treatments significantly associated with a higher risk of nasopharyngitis compared to placebo or other active treatments (**Tables S4**, **S5D**). However, it should be noted that despite the 95%CrI of dapagliflozin 10mg/d including the null value, the network results indicated a higher point estimate for the risk of nasopharyngitis when compared with placebo and other SGLT-2 inhibitors. According to the SUCRA values, dapagliflozin 10mg/d (81.6%) ranked first (**Table S6**). The comparison-adjusted funnel plot and Egger’s test (p=0.424) indicated no asymmetry (**Figure S3D**). There was no information to perform analyses of consistency.

#### 3.2.5 Bone fracture and amputation

Four RCTs involving 5,393 patients reported bone fracture (23, 25, 32, 43). Both the direct comparison and the synthesized results from the NMA did not show any treatments significantly associated with a higher risk of bone fracture compared to placebo or other active treatments (**Table S4**). However, it is important to note that this result is heavily influenced by one study that used canagliflozin 100mg/d, specifically the NCT02065791 trial (25). The pooled result remained stable after removing this trial. Insufficient information was available to perform analyses of consistency and publication bias. Additionally, only one trial included in our study reported amputation, involving 4,397 patients. The results showed no significant difference in the risk of lower limb amputation, with rates of 12.3 versus 11.2 per 1000 patient-years in the canagliflozin group and the placebo group, respectively (hazard ratio, 1.11; 95% CI, 0.79 to 1.56).

#### 3.2.6 Diabetic ketoacidosis

Two RCTs involving 4,705 patients reported DKA (23, 25). The synthesized results showed no significant difference between canagliflozin 100mg/d and placebo in terms of the risk of DKA (OR 3.45, 95%CrI 0.41 to 29.41) (**Table S4**). One trial with a large sample size from the NCT02065791 study reported low rates of DKA but a higher incidence in the canagliflozin 100mg/d group compared to the placebo group (2.2 vs. 0.2 per 1000 patient-years) (25). Another trial reported an incidence of DKA of 2.6% (4/154 patients) in the canagliflozin 100mg/d group and 1.9% (3/154 patients) in the placebo group (23).

#### 3.2.7 Renal-related AEs

Eight RCTs involving 6,883 patients reported renal-related AEs, with four trials specifically examining renal impairment in the context of dapagliflozin (21, 23, 25, 27, 31, 32, 42, 43). The direct comparison did not show any significant associations between treatments and a higher risk of renal-related AEs compared to placebo or other active treatments (**Table S4**). However, the synthesized results demonstrated that dapagliflozin 10mg/d (OR 30.82, 95% CrI 1.85 to 1682.39) and canagliflozin 100mg/d (OR 17.18, 95% CrI 1.05 to 1000.50) were significantly associated with a higher risk of renal-related AEs compared with metformin 2000mg/d, while no significant differences were observed between the other treatments (**Table S5E**). One trial with a large sample size from the NCT02065791 study reported similar but lower rates of AKI in the canagliflozin 100mg/d group compared to the placebo group (16.9 vs. 20.0 per 1000 patient-years, hazard ratio 0.85; 95% confidence interval [CI] 0.64 to 1.13). Based on the SUCRA values, dapagliflozin 10mg/d (80.7%) ranked first in terms of their association with renal-related AEs (**Table S6**).

### 3.3 Heterogeneity and subgroup analysis

The pairwise comparisons of heterogeneity in outcome estimates were presented in **Supplementary Material Table S4**, and it was observed that there were no significant heterogeneities between each treatment. According to the meta-regression results, the duration of trials did not significantly impact the risk of the adverse events of interest between interventions (**Table S8**). Subgroup analyses were conducted for gender in the UTI and GI outcomes (**Tables S9, S10**; **Figure S2**). Regarding UTI, the synthesized results of the NMA showed that no treatments were significantly associated with a higher risk of UTI compared to placebo or other active treatments, regardless of gender (**Table S9** and S10B). The SUCRA values indicated that dapagliflozin 10mg/d ranked first, with 90.8% in females and 79.4% in males, followed by dapagliflozin 5mg/d with 78.5% in females and 75.7% in males (**Table S11**). One trial that compared different dosages of dapagliflozin showed that the incidence of UTI was higher in the female group than the male group (dapagliflozin 10mg/d: 11.1% vs. 5.9%, dapagliflozin 5mg/d: 18.2% vs. 6.5%). Regarding GI, the synthesized results of the female subgroups showed that empagliflozin and dapagliflozin were associated with a higher risk of GI compared to placebo, with OR ranging between 6.21 (95% CrI 1.55 to 44.90) for empagliflozin 10mg/d and 20.18 (95% CrI 2.77 to 184.78) for dapagliflozin 10mg/d (**Table S10A**). However, in the synthesized results of the male subgroups, canagliflozin 100mg/d (OR 9.18, 95% CrI 1.77 to 53.68), canagliflozin 300mg/d (OR 18.84, 95% CrI 2.38 to 224.88), and empagliflozin 10mg/d (OR 5.38, 95% CrI 1.24 to 38.56) were associated with a higher risk of GI compared to placebo. It is important to note that the small number of included patients resulted in wide CrIs in most comparative analyses. Furthermore, the SUCRA values indicated that dapagliflozin 10mg/d (91.9%) and canagliflozin 300mg/d (88.8%) ranked first in the female and male subgroups, respectively (**Table S11**).

## 4 Discussion

This NMA of 10 pharmacological interventions from 25 double-blind trials, enrolling a total of 12,990 patients, and provides the comprehensive evidence with respect to the key safety outcomes associated with different recommended doses of SGLT-2i. Our study demonstrated that SGLT-2i do not appear to increased risk of DKA, nasopharyngitis, or bone fracture. Through direct comparison and the mixed treatment comparison, empagliflozin 10mg/d and canagliflozin 100mg/d were associated with the higher risk of GI and hypoglycemia, respectively. Moreover, dapagliflozin 10mg/d may be the riskiest according to the probability rankings for both UTI and renal impairment, however, the association for renal impairment with large uncertainty in the estimates owing to the small sample sizes of trials. Notably, this NMA and rankings have the potential to serve as a valuable decision-making tool for clinicians, facilitating informed treatment selection based on the safety profiles of different SGLT-2 inhibitors and their respective doses.

SGLT-2i are known for their ability to reduce hyperglycemia in patients by inhibiting renal glucose reabsorption in the proximal tubule of the kidney, leading to increased glucose excretion in the urine. However, this mechanism of action is also associated with an elevated risk of genital and urinary tract infections (45). Regarding UTI, our study revealed that only dapagliflozin at a dose of 10mg/d significantly increased the risk of UTI compared to other active SGLT-2i and placebo. This association was confirmed through overall synthesized results and gender subgroup analyses, particularly in females as indicated by SUCRA values. Although previous meta-analyses have shown no significant differences or only slight differences in UTI between patients using SGLT-2i and those taking placebo, subgroup analyses have consistently identified dapagliflozin 10mg/d as being associated with a higher risk of UTI, suggesting a dose-response relationship (12, 46–48). These findings are in line with previous studies and provide further support to our results. Additionally, retrospective studies on dapagliflozin 10mg/d discontinuation and hospitalization have reported UTI as a primary reason, with a significantly higher proportion of affected females (49, 50). In terms of GI, our study demonstrated that SGLT-2i had a greater association with GI risk compared to placebo and metformin 2000mg/d in patients with T2DM based on direct and network comparisons. However, no apparent differences were observed between lower and higher dosages of SGLT-2i. These findings were generally consistent with previous studies’ results (13, 48). Specifically, in the direct meta-analysis, dapagliflozin 10mg/d showed a higher risk of GI compared to dapagliflozin 5mg/d when compared to placebo. This correlation persisted in the network comparisons. This dose-response relationship is consistent with our previous finding for the risk of UTI with dapagliflozin, and one trial reported that dapagliflozin 10mg/d had a higher risk of GI compared to dapagliflozin 2.5mg/d (OR 1.55, 95% CI, 1.08 to 2.23) (51). Nevertheless, most genitourinary infections were typically mild to moderate in nature in our study, with a low rate of treatment discontinuation, and can be effectively resolved with routine antimicrobial therapy. Notably, our study found that empagliflozin 10mg/d ranked highest in terms of the probability of GI risk, which is consistent with the findings of a previous meta-analysis and a real-world analysis of the FAERS database (52, 53). Furthermore, an additional noteworthy finding in our study was that canagliflozin 300mg/d and dapagliflozin 10mg/d may be associated with the highest risk of GI in male and female subgroup analyses, respectively. However, due to limited up-to-date studies focusing on each gender, these conclusions should be interpreted cautiously, considering the sparse data available. Future well-designed trials with larger sample sizes are needed to validate these results. Considering the current evidence and the underlying mechanism of action of SGLT-2i, the potential risks of genitourinary infections should be carefully considered before initiating SGLT-2i therapy.

SGLT-2i have shown significant renal protective effects, including a 30% to 50% reduction in proteinuria and favorable outcomes in renal composite hard endpoints. The EMPA-REG OUTCOME trial demonstrated lower composite renal outcomes in patients treated with empagliflozin compared to placebo (HR = 0.68) (54). Similarly, the CREDENCE trial reported a reduced risk of composite renal outcomes in the canagliflozin group compared to placebo (HR = 0.70), with similar or lower rates of AKI in the canagliflozin 100mg/d group (HR = 0.85) (25). This systematic review highlights a lack of reporting on renal-related adverse events, with only 8 out of 25 randomized comparisons providing data, and only 4 reporting renal impairment related to dapagliflozin. Although the overall NMA suggests that dapagliflozin 10mg/d and canagliflozin 100mg/d may increase the risk of renal-related adverse events, the available evidence was insufficient to support or refute the potential risk of renal impairment or AKI specifically associated with the use of canagliflozin or dapagliflozin. Additionally, SGLT-2i medications may carry a risk of DKA by stimulating insulin release and promoting ketone reabsorption from the renal tubules, although the incidence of DKA is rare, approximately 0.1% (55). It has been suggested that the risk of DKA is negligible when the drug is properly prescribed (56). Similarly, due to their unique mechanism of action that is not dependent on promoting β-cell function or improving insulin resistance, SGLT-2i do not significantly increase the risk of hypoglycemia compared to placebo (57). Regarding nasopharyngitis, although no statistical differences were found between treatments in the studies reviewed, dapagliflozin 10mg/d may be associated with a higher risk of nasopharyngitis compared to placebo and other treatments. However, it is important to note that no clinical trials have been specifically conducted to evaluate this issue, and further data are needed to establish the true risk and determine if this is a class effect or specific to certain agents and dosages.

In 2016 and 2017, the FDA issued warnings regarding a potential increased risk of fractures and leg amputations with the use of canagliflozin (58, 59). However, the specific underlying mechanism leading to these risks associated with canagliflozin remains unknown. It has been suggested that SGLT-2i, by promoting glucosuria and volume depletion, may potentially reduce lower-limb tissue perfusion, which could play a role in the increased risk of fractures or amputations. Additionally, SGLT-2i increase serum phosphate levels by enhancing the tubular reabsorption of phosphate, and elevated phosphate levels can stimulate the release of parathyroid hormone, which may enhance bone resorption and increase the risk of fractures (60). However, recent meta-analyses have shown that neither the overall analysis nor subgroup analyses of SGLT-2i demonstrate a significant increased risk of fractures compared to other diabetes medications such as DPP-4 inhibitors, GLP-1 agonists, or placebo. Specifically, there is no evidence to suggest that individual SGLT-2i, including canagliflozin, dapagliflozin, and empagliflozin, at various doses were associated with an increased risk of bone fractures (61, 62). The results of the current study support the existing literature and demonstrate a neutral risk profile for fractures. In terms of amputation, based on the assessment of recent new clinical data and large-scale real-world studies, although the subgroup analyses suggest that the risk of amputations, although still increased with canagliflozin, is lower than previously described. Furthermore, overall analyses have not shown a significantly increased risk of amputations associated with SGLT-2i (63–67), thus, the boxed warning about e risk of amputations for canagliflozin was removed from the prescribing information by the FDA in 2020.

## 5 Limitation

This study has several limitations that should be taken into account. First, in order to directly observe the safety of SGLT-2i in patients with T2DM, we excluded the effects of combined with other drugs, and for that reason, the number of original trials included in this study was relatively small, and further confirmation of the findings are necessary. Second, the exclusion of all non-English and non-Chinese language literature may introduce potential publication bias. Third, certain outcomes may have been inadequately characterized within study or with wide 95% CrIs for OR values and imprecise estimates, primarily due to the limited number of studies available. Fourth, the specific types of infection between genital and urinary tract infections were not distinguished in this study, as very few of the included trials reported the specific types of infection in detail, this issue need more detailed clinical trials to address the data gap in the future.

## 6 Conclusion

In this NMA, current evidence from RCTs indicated that SGLT-2i were not significantly increased the risk of harm among comparison of different doses, except for dapagliflozin 10mg/d, which showed an increased risk of UTI and may be associated with a higher risk of renal impairment and nasopharyngitis. In terms of GI, empagliflozin 10mg/d, canagliflozin 300mg/d, and dapagliflozin 10mg/d were associated with a higher risk compared to placebo and metformin. Additionally, the evidence does not suggest a significantly increased risk of DKA, nasopharyngitis, and bone fracture with SGLT-2i, over placebo or active comparators. Further well-designed RCTs with larger sample sizes and more detailed information are required to verify and optimize the current body of evidence.

## Data availability statement

The original contributions presented in the study are included in the article/**Supplementary Material**. Further inquiries can be directed to the corresponding authors.

## Author contributions

LC: Conceptualization, Data curation, Formal Analysis, Investigation, Methodology, Software, Writing – original draft. QX: Conceptualization, Data curation, Formal Analysis, Methodology, Software, Writing – original draft. CY: Data curation, Formal Analysis, Software, Writing – review & editing. BT: Data curation, Formal Analysis, Writing – review & editing. LW: Data curation, Methodology, Writing – review & editing. BZ: Conceptualization, Supervision, Writing – review & editing. QZ: Conceptualization, Supervision, Writing – review & editing.
