# Supplementary material for: Comparative safety of different recommended doses of sodium–glucose cotransporter 2 inhibitors in patients with type 2 diabetes mellitus: a systematic review and network meta-analysis of randomized clinical trials
Source: Front Endocrinol (Lausanne). 2023 Nov 10;14:1256548. doi: 10.3389/fendo.2023.1256548 (PMC10667926; doi:10.3389/fendo.2023.1256548)
Supplement: Supplementary file 1 [file DataSheet_1.pdf]

Supplementary Material

Table S1 PRISMA NMA Checklist of Items to Include When Reporting a Systematic Review Involving a Network Meta-analysis

Section/Topic	Item #	Checklist Item	Reported on Page #
**TITLE**			
Title	1	Identify the report as a systematic review *incorporating a network meta-analysis (or related form of meta-analysis).*	1
**ABSTRACT**			
Structured summary	2	Provide a structured summary including, as applicable:<br>**Background:** main objectives<br>**Methods:** data sources; study eligibility criteria, participants, and interventions; study appraisal; and *synthesis methods, such as network meta-analysis.*<br>**Results:** number of studies and participants identified; summary estimates with corresponding confidence/credible intervals; *treatment rankings may also be discussed. Authors may choose to summarize pairwise comparisons against a chosen treatment included in their analyses for brevity.*<br>**Discussion/Conclusions:** limitations; conclusions and implications of findings.<br>**Other:** primary source of funding; systematic review registration number with registry name.	1-2
**INTRODUCTION**			
Rationale	3	Describe the rationale for the review in the context of what is already known, *including mention of why a network meta-analysis has been conducted.*	3
Objectives	4	Provide an explicit statement of questions being addressed, with reference to participants, interventions, comparisons, outcomes, and study design (PICOS).	3
**METHODS**			
Protocol and registration	5	Indicate whether a review protocol exists and if and where it can be accessed (e.g., Web address); and, if available, provide registration information, including registration number.	4

Eligibility criteria	6	Specify study characteristics (e.g., PICOS, length of follow-up) and report characteristics (e.g., years considered, language, publication status) used as criteria for eligibility, giving rationale. *Clearly describe eligible treatments included in the treatment network, and note whether any have been clustered or merged into the same node (with justification).*	4
Information sources	7	Describe all information sources (e.g., databases with dates of coverage, contact with study authors to identify additional studies) in the search and date last searched.	4
Search	8	Present full electronic search strategy for at least one database, including any limits used, such that it could be repeated.	Table S2
Study selection	9	State the process for selecting studies (i.e., screening, eligibility, included in systematic review, and, if applicable, included in the meta-analysis).	4
Data collection process	10	Describe method of data extraction from reports (e.g., piloted forms, independently, in duplicate) and any processes for obtaining and confirming data from investigators.	4
Data items	11	List and define all variables for which data were sought (e.g., PICOS, funding sources) and any assumptions and simplifications made.	4
**Geometry of the network**	**S1**	Describe methods used to explore the geometry of the treatment network under study and potential biases related to it. This should include how the evidence base has been graphically summarized for presentation, and what characteristics were compiled and used to describe the evidence base to readers.	5
Risk of bias within individual studies	12	Describe methods used for assessing risk of bias of individual studies (including specification of whether this was done at the study or outcome level), and how this information is to be used in any data synthesis.	5
Summary measures	13	State the principal summary measures (e.g., risk ratio, difference in means). *Also describe the use of additional summary measures assessed, such as treatment rankings and surface under the cumulative ranking curve (SUCRA) values, as well as modified approaches used to present summary findings from meta-analyses.*	5
Planned methods of analysis	14	Describe the methods of handling data and combining results of studies for each network meta-analysis. This should include, but not be limited to:<br>• *Handling of multi-arm trials;*<br>• *Selection of variance structure;*<br>• *Selection of prior distributions in Bayesian analyses; and*<br>• *Assessment of model fit.*	5
**Assessment of Inconsistency**	**S2**	Describe the statistical methods used to evaluate the agreement of direct and indirect evidence in the treatment network(s) studied. Describe efforts taken to address its presence when found.	5

Risk of bias across studies	15	Specify any assessment of risk of bias that may affect the cumulative evidence (e.g., publication bias, selective reporting within studies).	5
Additional analyses	16	Describe methods of additional analyses if done, indicating which were pre-specified. This may include, but not be limited to, the following:<br>• Sensitivity or subgroup analyses;<br>• Meta-regression analyses;<br>• *Alternative formulations of the treatment network; and*<br>• *Use of alternative prior distributions for Bayesian analyses (if applicable).*	5
**RESULTS†**			
Study selection	17	Give numbers of studies screened, assessed for eligibility, and included in the review, with reasons for exclusions at each stage, ideally with a flow diagram.	5
**Presentation of network structure**	**S3**	Provide a network graph of the included studies to enable visualization of the geometry of the treatment network.	6
**Summary of network geometry**	**S4**	Provide a brief overview of characteristics of the treatment network. This may include commentary on the abundance of trials and randomized patients for the different interventions and pairwise comparisons in the network, gaps of evidence in the treatment network, and potential biases reflected by the network structure.	5-6
Study characteristics	18	For each study, present characteristics for which data were extracted (e.g., study size, PICOS, follow-up period) and provide the citations.	Table 1
Risk of bias within studies	19	Present data on risk of bias of each study and, if available, any outcome level assessment.	Table S3
Results of individual studies	20	For all outcomes considered (benefits or harms), present, for each study: 1) simple summary data for each intervention group, and 2) effect estimates and confidence intervals. *Modified approaches may be needed to deal with information from larger networks.*	6-9
Synthesis of results	21	Present results of each meta-analysis done, including confidence/credible intervals. *In larger networks, authors may focus on comparisons versus a particular comparator (e.g. placebo or standard care), with full findings presented in an appendix. League tables and forest plots may be considered to summarize pairwise comparisons.* If additional summary measures were explored (such as treatment rankings), these should also be presented.	6-9<br>Table S4-S7

**Exploration for inconsistency**	**S5**	Describe results from investigations of inconsistency. This may include such information as measures of model fit to compare consistency and inconsistency models, *P* values from statistical tests, or summary of inconsistency estimates from different parts of the treatment network.	6-9<br>Table S7
Risk of bias across studies	22	Present results of any assessment of risk of bias across studies for the evidence base being studied.	Figure S2
Results of additional analyses	23	Give results of additional analyses, if done (e.g., sensitivity or subgroup analyses, meta-regression analyses, *alternative network geometries studied, alternative choice of prior distributions for Bayesian analyses,* and so forth).	8-9<br>Table S8-S10
**DISCUSSION**			
Summary of evidence	24	Summarize the main findings, including the strength of evidence for each main outcome; consider their relevance to key groups (e.g., healthcare providers, users, and policy-makers).	9-12
Limitations	25	Discuss limitations at study and outcome level (e.g., risk of bias), and at review level (e.g., incomplete retrieval of identified research, reporting bias). *Comment on the validity of the assumptions, such as transitivity and consistency. Comment on any concerns regarding network geometry (e.g., avoidance of certain comparisons).*	11
Conclusions	26	Provide a general interpretation of the results in the context of other evidence, and implications for future research.	12
**FUNDING**			
Funding	27	Describe sources of funding for the systematic review and other support (e.g., supply of data); role of funders for the systematic review. This should also include information regarding whether funding has been received from manufacturers of treatments in the network and/or whether some of the authors are content experts with professional conflicts of interest that could affect use of treatments in the network.	12

PICOS = population, intervention, comparators, outcomes, study design.

* Text in italics indicates wording specific to reporting of network meta-analyses that has been added to guidance from the PRISMA statement.

Table S2 Literature search strategy

PubMed

1	(((((((((((((((((((((Diabetes Mellitus, Type 2[MeSH Terms]) OR (Diabetes Mellitus, Type II[Title/Abstract])) OR (Type 2 Diabetes Mellitus[Title/Abstract])) OR (Type 2 Diabetes[Title/Abstract])) OR (Diabetes, Type 2[Title/Abstract])) OR (T2DM[Title/Abstract])) OR (Diabetes Mellitus, Noninsulin-Dependent[Title/Abstract])) OR (Diabetes Mellitus, Ketosis Resistant[Title/Abstract])) OR (Ketosis-Resistant Diabetes Mellitus[Title/Abstract])) OR (Diabetes Mellitus, Non Insulin Dependent[Title/Abstract])) OR (Non-Insulin-Dependent Diabetes Mellitus[Title/Abstract])) OR (Diabetes Mellitus, Stable[Title/Abstract])) OR (NIDDM[Title/Abstract])) OR (Diabetes Mellitus, Maturity-Onset[Title/Abstract])) OR (Diabetes Mellitus, Noninsulin Dependent[Title/Abstract])) OR (Maturity Onset Diabetes Mellitus[Title/Abstract])) OR (MODY[Title/Abstract])) OR (Diabetes Mellitus, Slow Onset[Title/Abstract])) OR (Noninsulin Dependent Diabetes Mellitus[Title/Abstract])) OR (Diabetes, Maturity-Onset[Title/Abstract])) OR (Diabetes Mellitus, Adult-Onset[Title/Abstract])) OR (Adult-Onset Diabetes Mellitus[Title/Abstract])
2	("Sodium-Glucose Transporter 2 Inhibitors"[MeSH Terms] OR " Sodium Glucose Transporter 2 Inhibitors"[Title/Abstract] OR "sodium-glucose cotransporter-2 inhibitor"[Title/Abstract] OR " Sodium-Glucose Transporter 2 Inhibitor"[Title/Abstract] OR "Sodium Glucose Transporter 2 Inhibitor"[Title/Abstract] OR "SGLT-2 Inhibitors"[Title/Abstract] OR “SGLT-2 Inhibitor” [Title/Abstract] OR "SGLT 2 Inhibitors"[Title/Abstract] OR SGLT 2 Inhibitor [Title/Abstract] OR "SGLT2 Inhibitors"[Title/Abstract] OR "SGLT2 Inhibitor"[Title/Abstract] OR “Inhibitor, SGLT2” [Title/Abstract] OR “Inhibitor, SGLT-2” [Title/Abstract] OR Gliflozins [Title/Abstract] OR Gliflozin [Title/Abstract] OR "Canagliflozin"[Mesh] OR Invokana[Title/Abstract]) OR (Canagliflozin Hemihydrate[Title/Abstract]) OR (Canagliflozin, Anhydrous[Title/Abstract]) OR dapagliflozin OR Farxiga[Title/Abstract] OR Forxiga[Title/Abstract] OR Empagliflozin OR Jardiance[Title/Abstract] OR Ipragliflozin OR Suglat[Title/Abstract] OR ASP1941[Title/Abstract] OR (Luseogliflozin) OR (Lusefi[Title/Abstract]) OR "1,5-anhydro-1-(5-(4-ethoxybenzyl)-2-methoxy-4-methylphenyl)-1-thioglucitol" [Supplementary Concept] OR (((((tofogliflozin hydrate[Title/Abstract]) OR (CSG452[Title/Abstract])) OR (tofogliflozin anhydrous[Title/Abstract])) OR (Apleway[Title/Abstract])) OR (Deberza[Title/Abstract])) OR (tofogliflozin) OR (ertugliflozin) OR (Steglatro[Title/Abstract])
3	("randomized controlled trial"[Publication Type] OR "randomized controlled trials as topic"[MeSH Terms] OR "randomized controlled trial"[All Fields] OR "randomised controlled trial"[All Fields] OR "randomized controlled trials as topic"[MeSH Terms] OR "controlled clinical trial"[Publication Type] OR "controlled clinical trials as topic"[MeSH Terms] OR "controlled clinical trial"[All Fields] OR "randomized"[Title/Abstract] OR "placebo"[Title/Abstract] OR "clinical trials as topic"[MeSH Major Topic] OR "randomly"[Title/Abstract] OR "trial"[Title/Abstract]) AND (humans[Filter])
	#1 AND #2 AND #3

Embase

#1	'non insulin dependent diabetes mellitus'/exp OR 'type 2 diabetes':ab,ti OR 'type 2 diabetes mellitus':ab,ti OR t2dm:ab,ti OR mody:ab,ti OR niddm:ab,ti OR 'ketosis-resistant diabetes mellitus':ab,ti OR 'adult onset diabetes mellitus':ab,ti OR 'noninsulin dependent diabetes mellitus':ab,ti OR 'maturity onset diabetes':ab,ti
#2	'sodium glucose cotransporter 2 inhibitor'/exp OR 'sodium-glucose cotransporter-2 inhibitor':ab,ti OR 'sodium-glucose cotransporter-2 inhibitors':ab,ti OR 'sodium-glucose transporter-2 inhibitors':ab,ti OR 'sodium glucose transporter 2 inhibitor':ab,ti OR 'sglt-2 inhibitors':ab,ti OR 'sglt-2 inhibitor':ab,ti OR 'sglt 2 inhibitors':ab,ti OR 'sglt 2 inhibitor':ab,ti OR 'canagliflozin'/exp OR 'canagliflozin':ab,ti OR 'invokana':ab,ti OR 'canagliflozin hemihydrate':ab,ti OR 'dapagliflozin'/exp OR farxiga:ab,ti OR forxiga:ab,ti OR 'empagliflozin'/exp OR jardiance:ab,ti OR 'ipragliflozin'/exp OR suglat:ab,ti OR 'luseogliflozin'/exp OR lusefi:ab,ti OR 'tofogliflozin anhydrous':ab,ti OR deberza:ab,ti OR 'tofogliflozin'/exp OR 'ertugliflozin'/exp OR 'steglatro':ab,ti OR dapagliflozin:ab,ti OR empagliflozin:ab,ti
#3	('randomized controlled trial'/exp OR ('randomized controlled trial'/exp AND topic) OR 'controlled clinical study'/exp OR randomy:ab,ti OR placebo:ab,ti OR randomized:ab,ti OR ('clinical trial'/exp AND topic) OR randomly:ab,ti OR trial:ab,ti OR blind*:ab,ti OR groups:ti,ab) NOT ('animal experiment'/exp NOT 'human'/exp)
	#1 AND #2 AND #3

Cochrane Library

#1	MeSH descriptor: [Diabetes Mellitus, Type 2] OR ((Type 2 Diabetes Mellitus):ti,ab,kw OR (Type 2 Diabetes):ti,ab,kw OR (T2DM):ti,ab,kw OR (Non-Insulin-Dependent Diabetes Mellitus):ti,ab,kw OR (Maturity Onset Diabetes Mellitus):ti,ab,kw) OR ((Stable Diabetes Mellitus):ti,ab,kw OR (Diabetes Mellitus, Type II):ti,ab,kw OR (MODY):ti,ab,kw OR (Slow-Onset Diabetes Mellitus):ti,ab,kw OR (Diabetes Mellitus, Ketosis Resistant):ti,ab,kw) OR ((Diabetes, Type 2):ti,ab,kw OR (Diabetes Mellitus, Type II):ti,ab,kw OR (NIDDM):ti,ab,kw)
#2	MeSH descriptor: [Sodium-Glucose Transporter 2 Inhibitors] OR ((SGLT 2 Inhibitor):ti,ab,kw OR (Sodium Glucose Transporter 2 Inhibitor):ti,ab,kw OR (Sodium-Glucose Transporter 2 Inhibitor):ti,ab,kw OR (SGLT-2 Inhibitor):ti,ab,kw OR (SGLT 2 Inhibitors):ti,ab,kw) OR ((SGLT 2 Inhibitor):ti,ab,kw OR (SGLT2 Inhibitor):ti,ab,kw OR (Inhibitor, SGLT-2):ti,ab,kw OR (SGLT2 Inhibitors):ti,ab,kw OR (Gliflozins):ti,ab,kw) OR (MeSH descriptor: [Sodium-Glucose Transport Proteins]) OR MeSH descriptor: [Canagliflozin] OR ((Canagliflozin Hemihydrate):ti,ab,kw OR (Invokana):ti,ab,kw OR (Canagliflozin, Anhydrous):ti,ab,kw) OR ((dapagliflozin):ti,ab,kw OR (Farxiga):ti,ab,kw OR (Forxiga):ti,ab,kw OR (Jardiance):ti,ab,kw OR (Suglat):ti,ab,kw) OR ((tofogliflozin hydrate):ti,ab,kw OR (tofogliflozin):ti,ab,kw OR (Deberza):ti,ab,kw OR (Apleway):ti,ab,kw OR ((ertugliflozin):ti,ab,kw OR (Steglatro):ti,ab,kw
#3	(randomized controlled trial):pt OR (controlled clinical trial):pt OR (random*):ti,ab,kw OR (placebo):ti,ab,kw OR (clinical trials):ti,ab,kw
	#1 AND #2 AND #3

Table S3. Results of risk of bias of included studies

Included trials	Randomization process	Deviations from intended interventions	Missing outcome data	Measurement of the outcome	Selection of the reported result	Overall Bias
List 2009	Some concerns	Low	Low	Low	Low	Some concerns
Roden 2015	Low	Low	Low	Low	Low	Low
Schwartz 2011	Some concerns	Low	Low	Low	Low	Some concerns
Kashiwagi 2014	Some concerns	Low	Low	Low	Low	Some concerns
Terra 2017	Some concerns	Low	Low	Low	Low	Some concerns
Iijima 2015	Some concerns	Low	Low	Low	Low	Some concerns
Stenlo¨f 2013	Some concerns	Low	Low	Low	Low	Some concerns
Hadjadj 2016	Low	Low	Low	Low	Low	Low
Rosenstock 2016	Some concerns	Low	Low	Low	Low	Some concerns
Lu 2021	Low	Low	Low	Low	Low	Low
Tikkanen 2015	Low	Low	Low	Low	Low	Low
Kadowaki 2014	Low	Low	Low	Low	Low	Low
Kaku 2014b	Low	Low	Low	Low	Low	Low
Seino 2014	Low	Low	Low	Low	Low	Low
Bailey 2015	Low	Low	Low	Low	Low	Low
Kaku 2014	Some concerns	Low	Low	Low	Low	Some concerns
Kaku 2013	Low	Low	Low	Low	Low	Low
Inagaki 2014	Low	Low	Low	Low	Low	Low
Inagaki 2013	Low	Low	Low	Low	Low	Low
Wada 2022	Some concerns	Low	Low	Low	Low	Some concerns
Ji 2014	Low	Low	Low	Low	Low	Low
Perkovic 2019	Low	Low	Low	Low	Low	Low
Ferrannini 2013	Low	Low	Low	Low	Low	Low
NCT00859898	Low	Low	Low	Low	Low	Low
NCT00643851	Low	Low	Low	Low	Low	Low

Note: The green background represents low risk of bias; The yellow background represents some concern

Table S4 Results of traditional pairwise meta-analysis of different safety outcomes

T1	T2	Genital infections				Unitary tract infections			
		Studies	Pairwise OR (95% Cl)	Heterogeneity		Studies	Pairwise OR (95% Cl)	Heterogeneity	
			Fixed effects	I^2	P-value		Fixed effects	I^2	P-value
Dapagliflozin 10mg/d	Dapagliflozin 5mg/d	5	1.53 (0.74, 3.16)	0.00%	0.921	5	1.19 (0.63, 2.23)	0.00%	0.686
Empagliflozin 10mg/d	Empagliflozin 25mg/d	5	1.07 (0.69, 1.68)	0.00%	0.896	5	0.95 (0.62, 1.43)	0.00%	0.995
Canagliflozin 100mg/d	Placebo	4	**3.66 (2.06, 6.50)**	0.00%	0.778	3	1.11 (0.92, 1.34)	0.00%	0.76
Dapagliflozin 10mg/d	Placebo	4	**7.33 (2.17, 24.73)**	0.00%	0.640	2	**2.14 (1.03, 4.44)**	0.00%	0.982
Dapagliflozin 5mg/d	Placebo	4	**4.47 (1.25, 15.92)**	0.00%	0.703	2	1.53 (0.76, 3.11)	0.00%	0.519
Empagliflozin 10mg/d	Placebo	4	**6.57 (2.53, 17.09)**	0.00%	0.567	4	0.92 (0.57, 1.50)	0.00%	0.975
Empagliflozin 25mg/d	Placebo	3	**6.62 (2.54, 17.21)**	0.00%	0.434	4	0.95 (0.59, 1.54)	0.00%	0.852
Canagliflozin 100mg/d	Canagliflozin 300mg/d	3	0.82 (0.47, 1.44)	0.00%	0.541	2	1.01 (0.52, 1.95)	0.00%	0.418
Canagliflozin 300mg/d	Placebo	1	3.04 (0.12, 75.83)	NA	NA	NR	NR	NR	NR
Ertugliflozin 15mg/d	Placebo	1	**4.23 (1.54, 11.64)**	NA	NA	1	0.44 (0.16, 1.20)	NA	NA
Ertugliflozin 5mg/d	Placebo	1	**2.92 (1.02, 8.31)**	NA	NA	1	0.82 (0.35, 1.89)	NA	NA
Ipragliflozin 100mg/d	Placebo	1	2.92 (0.12, 72.81)	NA	NA	2	1.72 (0.22, 13.34)	0.00%	0.554
Ipragliflozin 50mg/d	Placebo	1	4.93 (0.23, 104.54)	NA	NA	1	2.96 (0.3, 29.13)	NA	NA
Metformin 1500mg/d	Placebo	1	2.95 (0.12, 73.91)	NA	NA	1	1.67 (0.38, 7.34)	NA	NA
Ertugliflozin 15mg/d	Ertugliflozin 5mg/d	1	1.45 (0.70, 3.01)	NA	NA	1	0.54 (0.20, 1.50)	NA	NA
Ipragliflozin 100mg/d	Ipragliflozin 50mg/d	1	0.49 (0.04, 5.56)	NA	NA	2	0.71 (0.14, 3.69)	21.40%	0.259
Empagliflozin 10mg/d	Metformin 1000mg/d	1	2.85 (0.89, 9.14)	NA	NA	1	0.92 (0.42, 2.01)	NA	NA
Empagliflozin 25mg/d	Metformin 1000mg/d	1	2.10 (0.62, 7.11)	NA	NA	1	1.03 (0.47, 2.22)	NA	NA
Dapagliflozin 10mg/d	Metformin 1500mg/d	1	1.20 (0.07, 19.65)	NA	NA	1	1.21 (0.33, 4.48)	NA	NA
Dapagliflozin 5mg/d	Metformin 1500mg/d	1	0.96 (0.06, 15.81	NA	NA	1	0.96 (0.26, 3.52)	NA	NA
Canagliflozin 100mg/d	Metformin 2000mg/d	1	11.24 (0.62, 204.36)	NA	NA	1	1.00 (0.20, 5.01)	NA	NA
Canagliflozin 300mg/d	Metformin 2000mg/d	1	**21.83 (1.27, 374.65)**	NA	NA	1	1.67 (0.40, 7.08)	NA	NA
Dapagliflozin 10mg/d	Metformin 2000mg/d	1	**5.95 (2.25, 15.73)**	NA	NA	1	**2.72 (1.23, 6.00)**	NA	NA
Dapagliflozin 5mg/d	Metformin 2000mg/d	1	**3.65 (1.18, 11.28)**	NA	NA	1	1.06 (0.51, 2.21)	NA	NA

Empagliflozin 10mg/d	Metformin 2000mg/d	1	2.25 (0.77, 6.63)	NA	NA	1	0.74 (0.35, 1.57)	NA	NA
Empagliflozin 25mg/d	Metformin 2000mg/d	1	1.66 (0.53, 5.18)	NA	NA	1	0.82 (0.39, 1.73)	NA	NA
Metformin 1000mg/d	Metformin 2000mg/d	1	0.79 (0.21, 3.00)	NA	NA	1	0.80 (0.38, 1.69)	NA	NA
Canagliflozin 100mg/d	Sitagliptin 100mg/d	1	**3.80 (1.38, 10.46)**	NA	NA	1	1.34 (0.62, 2.92)	NA	NA
Canagliflozin 300mg/d	Sitagliptin 100mg/d	1	**3.76 (1.37, 10.34)**	NA	NA	1	1.15 (0.52, 2.55)	NA	NA
Empagliflozin 10mg/d	Sitagliptin 100mg/d	1	**6.81 (1.52, 30.53)**	NA	NA	1	1.05 (0.55, 2.00)	NA	NA
Empagliflozin 25mg/d	Sitagliptin 100mg/d	1	**7.40 (1.66, 32.96)**	NA	NA	1	1.00 (0.52, 1.92)	NA	NA
Placebo	Sitagliptin 100mg/d	1	1.96 (0.36, 10.83)	NA	NA	1	1.24 (0.67, 2.31)	NA	NA
Henagliflozin 10mg/d	Henagliflozin 5mg/d	NR	NR	NR	NR	1	0.71 (0.22, 2.27)	NA	NA
Henagliflozin 10mg/d	Placebo	NR	NR	NR	NR	1	0.83 (0.25, 2.79)	NA	NA
Henagliflozin 5mg/d	Placebo	NR	NR	NR	NR	1	1.18 (0.39, 3.60)	NA	NA

CONTINUE

T1	T2	**Hypoglycemia**				**Nasopharyngitis**			
			Pairwise OR (95%Cl)	Heterogeneity			Pairwise OR (95% CI)	Heterogeneity	
		Studies	Fixed effects	I^2	P-value	Studies	Fixed effects	I^2	P-value
Dapagliflozin 10mg/d	Dapagliflozin 5mg/d	4	1.10 (0.40, 3.04)	12.90%	0.328	4	1.54 (0.92, 2.56)	12.9%	0.328
Dapagliflozin 10mg/d	Placebo	4	0.83 (0.30, 2.26)	0.00%	0.958	4	1.28 (0.78, 2.10)	0.0%	0.642
Dapagliflozin 5mg/d	Placebo	4	0.80 (0.31, 2.08)	33.50%	0.211	4	0.82 (0.48, 1.39)	0.0%	0.444
Empagliflozin 10mg/d	Empagliflozin 25mg/d	4	1.00 (0.54, 1.84)	0.00%	0.921	4	0.98 (0.67, 1.44)	0.0%	0.526
Empagliflozin 10mg/d	Metformin 1000mg/d	1	3.00 (0.12, 74.16)	NA	NA	NR	NR	NR	NR
Empagliflozin 10mg/d	Metformin 2000mg/d	1	0.49 (0.04, 5.47)	NA	NA	NR	NR	NR	NR
Empagliflozin 25mg/d	Metformin 1000mg/d	1	3.09 (0.12, 76.39)	NA	NA	NR	NR	NR	NR
Empagliflozin 25mg/d	Metformin 2000mg/d	1	0.51 (0.05, 5.63)	NA	NA	NR	NR	NR	NR
Metformin 1000mg/d	Metformin 2000mg/d	1	0.20 (0.01, 4.12)	NA	NA	NR	NR	NR	NR
Canagliflozin 100mg/d	Canagliflozin 300mg/d	3	1.13 (0.58, 2.20)	0.00%	0.592	1	1.15 (0.44, 3.00)	NA	NA
Canagliflozin 100mg/d	Placebo	4	0.96 (0.80, 1.15)	0.00%	0.467	2	0.81 (0.34, 1.91)	26.9%	0.242

Canagliflozin 300mg/d	Placebo	1	3.04 (0.12, 75.83)	NA	NA	1	0.89 (0.34, 2.32)	NA	NA
Empagliflozin 10mg/d	Placebo	3	1.34 (0.67, 2.66)	0.00%	0.775	4	0.89 (0.61, 1.29)	16.8%	0.307
Empagliflozin 25mg/d	Placebo	3	1.33 (0.68, 2.61)	0.00%	0.852	4	0.90 (0.62, 1.31)	0.0%	0.784
Dapagliflozin 10mg/d	Metformin 1500mg/d	1	0.45 (0.08, 2.45)	NA	NA	NR	NR	NR	NR
Dapagliflozin 5mg/d	Metformin 1500mg/d	1	1.18 (0.34, 4.10)	NA	NA	NR	NR	NR	NR
Metformin 1500mg/d	Placebo	1	2.55 (0.47, 13.74)	NA	NA	NR	NR	NR	NR
Henagliflozin 10mg/d	Henagliflozin 5mg/d	1	2.03 (0.37, 11.24)	NA	NA	1	0.85 (0.28, 2.59)	NA	NA
Henagliflozin 10mg/d	Placebo	1	1.01 (0.25, 4.10)	NA	NA	1	0.66 (0.23, 1.89)	NA	NA
Henagliflozin 5mg/d	Placebo	1	0.50 (0.09, 2.75)	NA	NA	1	0.77 (0.28, 2.13)	NA	NA
Dapagliflozin 10mg/d	Metformin 2000mg/d	1	0.31 (0.06, 1.56)	NA	NA	NR	NR	NR	NR
Empagliflozin 10mg/d	Sitagliptin 100mg/d	1	1.00 (0.14, 7.13)	NA	NA	1	1.21 (0.70, 2.10)	NA	NA
Empagliflozin 25mg/d	Sitagliptin 100mg/d	1	1.00 (0.14, 7.16)	NA	NA	1	0.92 (0.51, 1.63)	NA	NA
Placebo	Sitagliptin 100mg/d	1	0.97 (0.14, 6.97)	NA	NA	1	0.97 (0.55, 1.71)	NA	NA
Canagliflozin 100mg/d	Metformin 2000mg/d	1	0.63 (0.24, 1.64)	NA	NA	NR	NR	NR	NR
Canagliflozin 300mg/d	Metformin 2000mg/d	1	0.81 (0.33, 1.99)	NA	NA	NR	NR	NR	NR
Canagliflozin 100mg/d	Sitagliptin 100mg/d	1	1.43 (0.53, 3.83)	NA	NA	NR	NR	NR	NR
Canagliflozin 300mg/d	Sitagliptin 100mg/d	1	0.97 (0.33, 2.83)	NA	NA	NR	NR	NR	NR
Ertugliflozin 15mg/d	Ertugliflozin 5mg/d	1	2.08 (0.38, 11.53)	NA	NA	NR	NR	NR	NR
Ertugliflozin 15mg/d	Placebo	1	2.04 (0.37, 11.31)	NA	NA	NR	NR	NR	NR
Ertugliflozin 5mg/d	Placebo	1	0.98 (0.14, 7.05)	NA	NA	NR	NR	NR	NR
Luseogliflozin 2.5mg/d	Luseogliflozin5mg/d	NR	NR	NR	NR	1	0.38 (0.07, 2.04)	NA	NA
Luseogliflozin 2.5mg/d	Placebo	NR	NR	NR	NR	1	0.42 (0.07, 2.41)	NA	NA
Luseogliflozin5mg/d	Placebo	NR	NR	NR	NR	1	1.12 (0.28, 4.39)	NA	NA
Placebo	Tofogliflozin 20mg/d	NR	NR	NR	NR	1	2.36 (0.82, 6.82)	NA	NA

CONTINUE

T1	T2	Bone fracture			
		Pairwise OR (95% Cl)		Heterogeneity	
		Studies	Fixed effects	I^2	P-value

Canagliflozin 100mg/d	Placebo	2	0.92 (0.66, 1.27)	41.00%	0.193
Dapagliflozin 10mg/d	Placebo	1	3.00 (0.12, 74.66)	NA	NA
Dapagliflozin 10mg/d	Metformin 2000mg/d	1	2.86 (0.12, 70.67)	NA	NA
Dapagliflozin 10mg/d	Dapagliflozin 5mg/d	1	2.97 (0.12, 73.81)	NA	NA
		**Renal-related AEs**			
T1	T2	Pairwise OR (95% Cl)		Heterogeneity	
		Studies	Fixed effects	I^2	P-value
Dapagliflozin 10mg/d	Placebo	3	2.04 (0.69, 6.04)	0.00%	0.551
Dapagliflozin 10mg/d	Dapagliflozin 5mg/d	3	3.30 (0.89, 12.17)	0.00%	0.760
Dapagliflozin 5mg/d	Placebo	2	0.60 (0.14, 2.57)	0.00%	0.864
Canagliflozin 100mg/d	Placebo	2	0.84 (0.71, 0.99)	0.00%	0.762
Luseogliflozin 2.5mg/d	Placebo	1	1.07 (0.31, 3.72)	NA	NA
Luseogliflozin 5.0mg/d	Placebo	1	1.07 (0.31, 3.72)	NA	NA
Luseogliflozin 2.5mg/d	Luseogliflozin 5.0mg/d	1	1.00 (0.30, 3.29)	NA	NA
Canagliflozin 300mg/d	Metformin 2000mg/d	1	7.06 (0.36, 137.42)	NA	NA
Canagliflozin 100mg/d	Metformin 2000mg/d	1	15.46 (0.88, 272.16)	NA	NA
Canagliflozin 100mg/d	Canagliflozin 300mg/d	1	2.38 (0.61, 9.33)	NA	NA
		**Diabetic ketoacidosis**			
T1	T2	Pairwise OR (95% Cl)			Heterogeneity
		Studies	Random effects	I^2	P-value
Canagliflozin 100mg/d	Placebo	2	3.45 (0.41, 29.41)	65.00%	0.090

Abbreviation: OR: odds ratio; Cl: confidence interval; NA: not applicable; NR: not reported; AEs: adverse events

Table S5A Results of Bayesian network meta-analysis of the genital infections outcome

Canagliflozin 100mg/d														
0.76<br>(0.37, 1.43)	Canagliflozin 300mg/d													
0.51<br>(0.16, 1.40)	0.68<br>(0.19, 2.14)	Empagliflozin 10mg/d												
0.56<br>(0.18, 1.55)	0.74<br>(0.21, 2.38)	1.09<br>(0.65, 1.93)	Empagliflozin 25mg/d											
1.20<br>(0.35, 3.75)	1.58<br>(0.43, 5.79)	2.33<br>(0.63, 9.21)	2.13<br>(0.56, 8.43)	Dapagliflozin 5mg/d										
0.78<br>(0.23, 2.55)	1.02<br>(0.29, 3.68)	1.53<br>(0.42, 5.64)	1.41<br>(0.39, 5.15)	0.65<br>(0.31, 1.36)	Dapagliflozin 10mg/d									
1.30<br>(0.31, 5.13)	1.71<br>(0.38, 7.90)	2.57<br>(0.55, 11.93)	2.36<br>(0.50, 11.08)	1.10<br>(0.22, 5.80)	1.70<br>(0.34, 8.04)	Ertugliflozin 5mg/d								
0.90<br>(0.21, 3.40)	1.18<br>(0.26, 5.40)	1.76<br>(0.37, 7.99)	1.62<br>(0.34, 7.17)	0.75<br>(0.15, 3.62)	1.15<br>(0.24, 5.32)	0.69<br>(0.27, 1.70)	Ertugliflozin 15mg/d							
0.00<br>(0.00, 9.00)	0.00<br>(0.00, 12.46)	0.00<br>(0.00, 18.69)	0.00<br>(0.00, 16.61)	0.00<br>(0.00, 7.60)	0.00<br>(0.00, 12.47)	0.00<br>(0.00, 5.97)	0.00<br>(0.00, 8.87)	Ipragliflozin 100mg/d						
0.00<br>(0.00, 1.45)	0.00<br>(0.00, 1.91)	0.00<br>(0.00, 3.11)	0.00<br>(0.00, 3.13)	0.00<br>(0.00, 1.35)	0.00<br>(0.00, 1.95)	0.00<br>(0.00, 1.05)	0.00<br>(0.00, 1.67)	0.39<br>(0.01, 5.40)	Ipragliflozin 50mg/d					
**3.75<br>(1.49, 10.77)**	**4.95<br>(1.93, 15.04)**	**7.33<br>(2.55, 27.13)**	**6.72<br>(2.33, 23.95)**	3.15<br>(0.80, 14.08)	**4.82<br>(1.29, 20.87)**	2.94<br>(0.61, 15.38)	4.24<br>(0.91, 23.10)	—	—	Sitagliptin 100mg/d				
1.41<br>(0.29, 7.51)	1.88<br>(0.35, 10.94)	2.79<br>(0.77, 11.23)	2.54<br>(0.70, 10.42)	1.19<br>(0.17, 7.69)	1.83<br>(0.26, 11.50)	1.07<br>(0.17, 8.18)	1.56<br>(0.25, 11.83)	—	—	0.37<br>(0.07, 2.11)	Metformin 1000mg/d			
1.06<br>(0.26, 4.42)	1.40<br>(0.32, 6.47)	2.04<br>(0.75, 6.62)	1.89<br>(0.67, 5.99)	0.89<br>(0.19, 4.32)	1.35<br>(0.32, 6.42)	0.80<br>(0.15, 5.07)	1.17<br>(0.21, 7.52)	—	—	0.28<br>(0.06, 1.22)	0.74<br>(0.17, 3.15)	Metformin 1500mg/d		
**5.81<br>(1.75, 20.82)**	**7.62<br>(2.17, 31.28)**	**11.39<br>(2.95, 55.20)**	**10.44<br>(2.69, 45.39)**	**4.91<br>(1.98, 13.19)**	**7.49<br>(3.27, 19.46)**	4.45<br>(0.89, 26.03)	**6.50<br>(1.34, 37.57)**	—	—	1.56<br>(0.34, 6.83)	4.15<br>(0.62, 33.06)	**5.60<br>(1.06, 32.17)**	Metformin 2000mg/d	
**4.02<br>(2.09, 8.12)**	**5.33<br>(2.25, 13.83)**	**7.90<br>(3.39, 22.08)**	**7.22<br>(3.11, 20.04)**	**3.36<br>(1.21, 10.13)**	**5.16<br>(1.90, 15.97)**	3.11<br>(0.97, 11.05)	**4.48<br>(1.44, 16.22)**	—	—	1.09<br>(0.35, 2.91)	2.88<br>(0.58, 12.91)	**3.89<br>(1.01, 13.85)**	0.69<br>(0.20, 2.18)	Placebo

Effect sizes represent summary odds ratio and 95% credible intervals. “-” indicate the effect size with very width credible interval owing to the small sample sizes, which were not shown. OR>1 indicate that the treatment specified in the column got more risk effect than that specified in the row, which values with significant differences are in bold.

Table S5B Results of Bayesian network meta-analysis of the urinary tract infections outcome

Canagliflozin 100mg/d																
1.00 (0.55, 1.86)	Canagliflozin 300mg/d															
**0.52 (0.29, 0.95)**	0.52 (0.23, 1.18)	Dapagliflozin 10mg/d														
0.77 (0.41, 1.43)	0.77 (0.33, 1.77)	1.47 (0.85, 2.58)	Dapagliflozin 5mg/d													
1.26 (0.80, 1.99)	1.26 (0.61, 2.55)	**2.40 (1.26, 4.64)**	1.63 (0.84, 3.18)	Empagliflozin 10mg/d												
1.19 (0.76, 1.88)	1.19 (0.58, 2.41)	**2.27 (1.20, 4.36)**	1.54 (0.79, 3.00)	0.95 (0.62, 1.44)	Empagliflozin 25mg/d											
2.59 (0.96, 7.91)	2.61 (0.81, 9.11)	**4.97 (1.59, 17.09)**	**3.38 (1.07, 11.72)**	2.07 (0.71, 6.7)	2.18 (0.75, 7.05)	Ertugliflozin 15mg/d										
1.36 (0.57, 3.30)	1.36 (0.48, 3.94)	2.6 (0.94, 7.39)	1.77 (0.63, 5.06)	1.08 (0.42, 2.84)	1.15 (0.45, 3.00)	0.53 (0.17, 1.44)	Ertugliflozin 5mg/d									
1.35 (0.38, 4.96)	1.35 (0.33, 5.61)	2.57 (0.66, 10.5)	1.75 (0.44, 7.24)	1.07 (0.29, 4.16)	1.13 (0.30, 4.40)	0.52 (0.10, 2.61)	0.99 (0.22, 4.58)	Henagliflozin 10mg/d								
0.93 (0.29, 2.95)	0.93 (0.25, 3.40)	1.77 (0.49, 6.35)	1.21 (0.32, 4.37)	0.74 (0.21, 2.50)	0.78 (0.23, 2.63)	0.36 (0.07, 1.61)	0.68 (0.16, 2.81)	0.69 (0.20, 2.26)	Henagliflozin 5mg/d							
0.47 (0.01, 6.07)	0.46 (0.01, 6.52)	0.89 (0.03, 12.3)	0.60 (0.02, 8.32)	0.37 (0.01, 4.97)	0.39 (0.01, 5.25)	0.17 (0.00, 2.79)	0.34 (0.01, 5.10)	0.34 (0.01, 5.96)	0.49 (0.01, 8.34)	Ipragliflozin 100mg/d						
0.29 (0.01, 2.79)	0.29 (0.01, 3.02)	0.55 (0.02, 5.72)	0.37 (0.01, 3.90)	0.23 (0.01, 2.29)	0.24 (0.01, 2.41)	0.11 (0.00, 1.32)	0.21 (0.01, 2.38)	0.21 (0.01, 2.86)	0.30 (0.01, 3.97)	0.62 (0.07, 4.25)	Ipragliflozin 50mg/d					
1.24 (0.60, 2.68)	1.24 (0.50, 3.17)	**2.37 (1.06, 5.55)**	1.61 (0.71, 3.80)	0.99 (0.50, 2.03)	1.04 (0.53, 2.14)	0.48 (0.13, 1.65)	0.91 (0.30, 2.82)	0.92 (0.21, 3.92)	1.34 (0.35, 5.34)	2.71 (0.19, 91.34)	4.37 (0.40, 140.32)	Metformin 1000mg/d				
0.70 (0.22, 2.50)	0.70 (0.20, 2.8)	1.34 (0.45, 4.62)	0.91 (0.30, 3.15)	0.56 (0.17, 2.06)	0.59 (0.18, 2.17)	0.27 (0.06, 1.33)	0.52 (0.12, 2.34)	0.53 (0.09, 3.00)	0.76 (0.15, 4.14)	1.56 (0.09, 58.86)	2.53 (0.19, 91.41)	0.57 (0.15, 2.30)	Metformin 1500mg/d			
1.05 (0.61, 1.81)	1.05 (0.49, 2.23)	**2.00 (1.18, 3.44)**	1.36 (0.79, 2.35)	0.83 (0.48, 1.44)	0.88 (0.51, 1.52)	0.40 (0.12, 1.22)	0.77 (0.28, 2.08)	0.78 (0.19, 3.00)	1.13 (0.32, 4.05)	2.27 (0.17, 73.98)	3.66 (0.35, 114.48)	0.85 (0.40, 1.69)	1.50 (0.42, 4.66)	Metformin 2000mg/d		
1.28 (0.80, 2.09)	1.28 (0.66, 2.48)	**2.44 (1.21, 5.02)**	1.66 (0.81, 3.46)	1.02 (0.61, 1.73)	1.08 (0.64, 1.83)	0.49 (0.15, 1.46)	0.94 (0.35, 2.47)	0.95 (0.24, 3.61)	1.38 (0.40, 4.87)	2.77 (0.20, 89.69)	4.47 (0.44, 138.78)	1.03 (0.46, 2.27)	1.82 (0.48, 6.13)	1.22 (0.65, 2.34)	Sitagliptin 100mg/d	
1.11 (0.92, 1.33)	1.11 (0.59, 2.06)	**2.11 (1.20, 3.79)**	1.44 (0.79, 2.63)	0.88 (0.57, 1.35)	0.93 (0.61, 1.42)	0.43 (0.14, 1.13)	0.81 (0.34, 1.89)	0.82 (0.23, 2.86)	1.19 (0.38, 3.83)	2.38 (0.18, 75.56)	3.84 (0.40, 118.10)	0.89 (0.42, 1.81)	1.58 (0.45, 4.88)	1.06 (0.63, 1.78)	0.87 (0.54, 1.38)	Placebo

Effect sizes represent summary odds ratio and 95% credible intervals. OR>1 indicate that the treatment specified in the column got more risk effect than that specified in the row, which values with significant differences are in bold.

Table S5C Results of Bayesian network meta-analysis of the hypoglycemia outcomes

Canagliflozin 100mg/d														
1.23 (0.35, 4.40)	Canagliflozin 300mg/d													
2.22 (0.46, 11.18)	1.82 (0.29, 11.82)	Empagliflozin 10mg/d												
1.86 (0.37, 8.58)	1.52 (0.24, 9.18)	0.84 (0.20, 3.19)	Empagliflozin 25mg/d											
5.18 (1.00, 33.20)	4.26 (0.60, 35.89)	2.33 (0.33, 19.13)	2.79 (0.42, 23.64)	Dapagliflozin 5mg/d										
3.72 (0.82, 17.24)	3.04 (0.49, 18.61)	1.66 (0.26, 10.45)	2.01 (0.33, 12.68)	0.71 (0.14, 3.06)	Dapagliflozin 10mg/d									
3.27 (0.16, 67.84)	2.67 (0.10, 66.63)	1.47 (0.06, 35.78)	1.76 (0.07, 43.45)	0.62 (0.02, 14.99)	0.88 (0.04, 20.34)	Ertugliflozin 5mg/d								
1.39 (0.08, 20.69)	1.14 (0.05, 20.61)	0.63 (0.03, 10.75)	0.75 (0.04, 13.36)	0.27 (0.01, 4.48)	0.38 (0.02, 6.24)	0.43 (0.03, 5.27)	Ertugliflozin 15mg/d							
7.20 (0.49, 131.54)	5.88 (0.32, 126.73)	3.27 (0.18, 67.71)	3.90 (0.22, 84.62)	1.39 (0.07, 28.20)	1.94 (0.11, 38.55)	2.22 (0.05, 115.41)	5.27 (0.15, 231.15)	Henagliflozin 5mg/d						
3.14 (0.24, 38.62)	2.57 (0.16, 39.21)	1.42 (0.09, 20.93)	1.68 (0.11, 26.36)	0.60 (0.03, 8.58)	0.85 (0.06, 11.87)	0.95 (0.02, 39.75)	2.25 (0.07, 80.14)	0.44 (0.03, 5.40)	Henagliflozin 10mg/d					
—	—	—	—	—	—	—	—	—	—	Metformin 1000mg/d				
2.54 (0.27, 24.26)	2.07 (0.18, 24.89)	1.14 (0.10, 13.49)	1.37 (0.12, 16.73)	0.48 (0.06, 3.65)	0.68 (0.09, 5.40)	0.78 (0.02, 26.69)	1.84 (0.07, 53.65)	0.35 (0.01, 9.09)	0.81 (0.04, 18.99)	0.00 (0.00, 0.53)	Metformin 1500mg/d			
0.87 (0.20, 3.66)	0.71 (0.14, 3.50)	0.39 (0.06, 2.22)	0.47 (0.08, 2.76)	0.17 (0.02, 1.09)	0.23 (0.04, 1.18)	0.27 (0.01, 6.78)	0.62 (0.03, 13.44)	0.12 (0.01, 2.19)	0.28 (0.02, 4.41)	0.00 (0.00, 0.15)	0.34 (0.03, 3.75)	Metformin 2000mg/d		
1.64 (0.36, 7.97)	1.34 (0.26, 7.36)	0.73 (0.12, 4.69)	0.88 (0.15, 5.87)	0.32 (0.03, 2.61)	0.44 (0.06, 3.23)	0.50 (0.02, 13.60)	1.19 (0.06, 28.83)	0.23 (0.01, 4.59)	0.53 (0.03, 9.12)	0.00 (0.00, 0.32)	0.65 (0.05, 8.52)	1.91 (0.29, 13.37)	Sitagliptin 100mg/d	
**3.12 (1.18, 8.36)**	2.55 (0.60, 10.93)	1.40 (0.34, 5.65)	1.68 (0.43, 6.92)	0.60 (0.12, 2.43)	0.84 (0.23, 3.02)	0.96 (0.05, 16.71)	2.24 (0.18, 33.45)	0.43 (0.03, 5.42)	1.00 (0.10, 10.47)	0.00 (0.00, 0.55)	1.23 (0.16, 9.26)	3.61 (0.82, 15.87)	1.90 (0.37, 9.15)	Placebo

Effect sizes represent summary odds ratio and 95% credible intervals. "-" indicate the effect size with very width credible interval owing to the small sample sizes, which were not shown. OR>1 indicate that the treatment specified in the column got more risk effect than that specified in the row, which values with significant differences are in bold.

Table S5D Results of Bayesian network meta-analysis of the nasopharyngitis outcomes

Canagliflozin 100mg/d												
1.02 (0.32, 3.05)	Canagliflozin 300mg/d											
0.86 (0.26, 2.70)	0.86 (0.25, 2.84)	Empagliflozin 10mg/d										
0.83 (0.26, 2.59)	0.82 (0.23, 2.64)	0.95 (0.56, 1.58)	Empagliflozin 25mg/d									
0.92 (0.26, 3.09)	0.91 (0.25, 3.15)	1.06 (0.46, 2.36)	1.10 (0.50, 2.47)	Dapagliflozin 5mg/d								
0.60 (0.18, 1.90)	0.59 (0.17, 2.09)	0.69 (0.31, 1.49)	0.71 (0.34, 1.58)	0.66 (0.35, 1.17)	Dapagliflozin 10mg/d							
1.02 (0.20, 4.97)	1.02 (0.20, 5.21)	1.18 (0.32, 4.14)	1.23 (0.34, 4.40)	1.12 (0.28, 4.15)	1.78 (0.44, 6.29)	Henagliflozin 5mg/d						
1.19 (0.23, 6.02)	1.14 (0.23, 6.71)	1.31 (0.39, 5.36)	1.38 (0.41, 5.63)	1.27 (0.33, 5.21)	1.99 (0.52, 7.62)	1.19 (0.32, 4.46)	Henagliflozin 10mg/d					
1.85 (0.24, 18.72)	1.84 (0.23, 20.44)	2.09 (0.33, 18.13)	2.20 (0.34, 18.47)	1.96 (0.32, 18.27)	3.00 (0.53, 27.99)	1.81 (0.23, 22.08)	1.47 (0.19, 17.54)	Luseogliflozin 2.5mg/d				
0.65 (0.10, 4.54)	0.63 (0.10, 4.59)	0.79 (0.14, 3.65)	0.82 (0.15, 3.87)	0.74 (0.14, 3.50)	1.12 (0.22, 5.50)	0.66 (0.09, 4.19)	0.59 (0.07, 3.70)	0.37 (0.04, 2.04)	Luseogliflozin 5mg/d			
1.93 (0.36, 9.37)	1.91 (0.34, 10.75)	2.19 (0.61, 8.59)	2.28 (0.64, 8.88)	2.07 (0.56, 8.13)	3.18 (0.87, 13.42)	1.91 (0.36, 10.80)	1.62 (0.30, 8.66)	1.05 (0.09, 8.87)	2.92 (0.42, 21.67)	Tofogliflozin 20mg/d		
0.83 (0.23, 2.94)	0.84 (0.21, 3.13)	0.97 (0.47, 2.01)	1.01 (0.50, 2.17)	0.91 (0.36, 2.44)	1.42 (0.56, 3.62)	0.83 (0.21, 3.38)	0.71 (0.16, 2.89)	0.47 (0.05, 3.32)	1.22 (0.25, 7.70)	0.44 (0.11, 1.77)	Sitagliptin 100mg/d	
0.78 (0.26, 2.09)	0.76 (0.24, 2.19)	0.89 (0.52, 1.43)	0.94 (0.57, 1.52)	0.85 (0.46, 1.53)	1.30 (0.72, 2.28)	0.74 (0.23, 2.49)	0.66 (0.18, 2.13)	0.43 (0.05, 2.46)	1.18 (0.26, 5.68)	0.41 (0.11, 1.29)	0.92 (0.43, 1.86)	Placebo

Effect sizes represent summary odds ratio and 95% credible intervals. OR>1 indicate that the treatment specified in the column got more risk effect than that specified in the row.

Table S5E Results of Bayesian network meta-analysis of the renal-related adverse events outcomes

Canagliflozin 100mg/d							
2.11 (0.11, 26.42)	Canagliflozin 300mg/d						
2.06 (0.25, 46.19)	1.00 (0.05, 104.56)	Dapagliflozin 5mg/d					
0.55 (0.07, 6.48)	0.25 (0.01, 13.35)	0.25 (0.03, 1.41)	Dapagliflozin 10mg/d				
0.99 (0.06, 29.40)	0.46 (0.01, 42.69)	0.45 (0.01, 10.74)	1.78 (0.09, 47.99)	Luseogliflozin 2.5mg/d			
0.99 (0.06, 31.86)	0.46 (0.01, 43.40)	0.46 (0.01, 10.57)	1.77 (0.09, 42.30)	1.03 (0.08, 11.64)	Luseogliflozin5mg/d		
**17.18 (1.05, 1000.50)**	9.13 (0.31, 1034.47)	7.84 (0.24, 468.96)	**30.82 (1.85, 1682.39)**	18.02 (0.31, 2040.92)	17.58 (0.35, 1874.60)	Metformin 2000mg/d	
1.00 (0.27, 8.67)	0.49 (0.04, 22.63)	0.49 (0.05, 3.17)	1.94 (0.39, 12.87)	1.05 (0.08, 14.69)	1.07 (0.08, 14.20)	0.06 (0.00, 1.25)	Placebo

Effect sizes represent summary odds ratio and 95% credible intervals. OR>1 indicate that the treatment specified in the column got more risk effect than that specified in the row, which values with significant differences are in bold.

Table S6 SUCRA values of treatments modalities under different endpoint safety outcomes

Treatments	SUCRA values				
	**UTI**	**GI**	**Hypoglycemia**	**Nasopharyngitis**	**Renal-related AEs**
Empagliflozin 10mg/d	33.2	**75.9**	52.7	53.1	NA
Empagliflozin 25mg/d	38.3	72.2	59.2	57.7	NA
Canagliflozin 300mg/d	52.6	60.7	72.1	47.0	38.6
Canagliflozin 100mg/d	55.7	47.9	**80.6**	46.1	59.4
Dapagliflozin 10mg/d	**87.2**	60.4	36.7	**81.6**	**80.7**
Dapagliflozin 5mg/d	70.1	42.5	27.5	48.8	34.5
Ertugliflozin 15mg/d	7.5	54.0	66.2	NA	NA
Ertugliflozin 5mg/d	32.5	39.3	45	NA	NA
Ipragliflozin 50mg/d	83.6	**95.7**	NA	NA	NA
Ipragliflozin 100mg/d	69.7	89.9	NA	NA	NA
Henagliflozin 5mg/d	55.3	NA	27.4	46.7	NA
Henagliflozin 10mg/d	35.6	NA	45.6	37.6	NA
Luseogliflozin 2.5mg/d	NA	NA	NA	23.7	60.8
Luseogliflozin 5mg/d	NA	NA	NA	66.4	60.8
Tofogliflozin 20mg/d	NA	NA	NA	19.0	NA
Metformin 1500mg/d	67.7	46.8	50.1	NA	NA
Metformin 1000mg/d	36.5	38.2	1.5	NA	NA
Metformin 2000mg/d	48.6	5.2	**82.7**	NA	NA
Sitagliptin 100mg/d	32.2	11.7	62.3	56.0	5.7
Placebo	43.6	9.6	40.4	66.3	59.5

Note: SUCRA: surface under the cumulative ranking curves; UTI: urinary tract infection, GI: genital infection, NA: not applicable

Table S7A Node-splitting analysis of the genital infection outcome

Name	Direct Effect	Indirect Effect	Overall	P-Value
Canagliflozin 100mg/d, Sitagliptin 100mg/d	-1.25 (-2.54, -0.10)	-1.71 (-4.11, 0.11)	-1.32 (-2.38, -0.40)	0.69
Canagliflozin 100mg/d, Metformin 2000mg/d	-28.12 (-94.20, -3.42)	-1.08 (-2.56, 0.74)	-1.66 (-3.26, -0.50)	**0.00**
Canagliflozin 100mg/d, Placebo	-1.27 (-2.05, -0.51)	-2.00 (-3.83, -0.46)	-1.39 (-2.09, -0.74)	0.46
Canagliflozin 300mg/d, Sitagliptin 100mg/d	-1.52 (-2.77, -0.35)	-2.00 (-4.19, -0.03)	-1.60 (-2.71, -0.66)	0.66
Canagliflozin 300mg/d, Metformin 2000mg/d	-30.65 (-89.68, -3.69)	-1.31 (-2.95, 0.54)	-1.98 (-3.73, -0.76)	**0.00**
Canagliflozin 300mg/d, Placebo	-32.04 (-58.98, -1.02)	-1.66 (-2.62, -0.69)	-1.67 (-2.63, -0.81)	0.07
Empagliflozin 10mg/d, Sitagliptin 100mg/d	-2.25 (-4.19, -0.75)	-1.84 (-3.58, -0.35)	-1.99 (-3.30, -0.94)	0.72
Empagliflozin 10mg/d, Metformin 1500mg/d	-0.80 (-2.18, 0.38)	-0.74 (-4.39, 2.21)	-0.71 (-1.89, 0.28)	0.99
Empagliflozin 10mg/d, Placebo	-2.02 (-3.07, -1.02)	-2.33 (-3.93, -0.62)	-2.07 (-3.09, -1.22)	0.72
Empagliflozin 25mg/d, Sitagliptin 100mg/d	-2.03 (-4.06, -0.57)	-1.41 (-3.55, 0.03)	-1.90 (-3.18, -0.85)	0.57
Empagliflozin 25mg/d, Metformin 1500mg/d	-0.66 (-1.94, 0.49)	-0.42 (-4.00, 2.37)	-0.64 (-1.79, 0.40)	0.89
Empagliflozin 25mg/d, Placebo	-1.90 (-3.15, -0.87)	-2.35 (-4.09, -0.53)	-1.98 (-3.00, -1.13)	0.68
Dapagliflozin 5mg/d, Dapagliflozin 10mg/d	0.45 (-0.32, 1.29)	-0.09 (-1.61, 1.70)	0.43 (-0.31, 1.19)	0.53
Dapagliflozin 5mg/d, Metformin 1500mg/d	0.03 (-3.35, 2.47)	0.11 (-1.80, 1.91)	0.12 (-1.46, 1.66)	0.96
Dapagliflozin 5mg/d, Metformin 2000mg/d	-1.25 (-2.96, -0.01)	-1.82 (-3.44, -0.46)	-1.59 (-2.58, -0.69)	0.61
Dapagliflozin 5mg/d, Placebo	-1.64 (-3.19, -0.34)	-0.01 (-2.03, 2.39)	-1.21 (-2.32, -0.19)	0.15
Dapagliflozin 10mg/d, Metformin 1500mg/d	-0.20 (-3.69, 2.04)	-0.33 (-2.18, 1.44)	-0.30 (-1.86, 1.15)	0.94
Dapagliflozin 10mg/d, Metformin 2000mg/d	-1.79 (-3.15, -0.75)	-2.42 (-4.20, -0.93)	-2.01 (-2.97, -1.18)	0.55
Dapagliflozin 10mg/d, Placebo	-2.13 (-3.73, -0.98)	-0.24 (-2.62, 2.12)	-1.64 (-2.77, -0.64)	0.21
Sitagliptin 100mg/d, Placebo	0.86 (-1.30, 2.52)	-0.42 (-1.53, 0.91)	-0.09 (-1.07, 1.04)	0.23
Metformin 1500mg/d, Placebo	-27.80 (-95.21, -1.70)	-1.28 (-2.57, 0.03)	-1.37 (-2.63, -0.15)	**0.04**

P-values < 0.05 are in bold, indicating a significant inconsistency between the direct effect and indirect effects.

Table S7B Node-splitting analysis of the urinary tract infection outcome

Name	Direct Effect	Indirect Effect	Overall	P-Value
Canagliflozin 100mg/d, Metformin 2000mg/d	-0.014 (-1.7, 1.8)	0.017 (-0.67, 0.72)	-0.044 (-0.65, 0.58)	0.97
Canagliflozin 100mg/d, Placebo	-0.083 (-0.44, 0.36)	-0.18 (-1.1, 0.76)	-0.096 (-0.41, 0.27)	0.84
Canagliflozin 100mg/d, Sitagliptin 100mg/d	-0.30 (-1.2, 0.57)	-0.24 (-1.0, 0.55)	-0.24 (-0.79, 0.34)	0.91
Canagliflozin 300mg/d, Metformin 2000mg/d	-0.55 (-2.2, 0.97)	0.16 (-0.85, 1.2)	-0.069 (-0.87, 0.74)	0.44
Canagliflozin 300mg/d, Sitagliptin 100mg/d	-0.14 (-1.0, 0.76)	-0.76 (-2.2, 0.76)	-0.26 (-0.99, 0.47)	0.48
Dapagliflozin 10mg/d, Dapagliflozin 5mg/d	-0.19 (-0.88, 0.47)	-0.96 (-2.2, 0.26)	-0.39 (-0.97, 0.19)	0.28
Dapagliflozin 10mg/d, Metformin 2000mg/d	-1.0 (-2., -0.19)	-0.39 (-1.2, 0.41)	-0.70 (-1.3, -0.13)	0.28
Dapagliflozin 10mg/d, Placebo	-0.62 (-1.4, 0.13)	-1.1 (-2.3, 0.0037)	-0.74 (-1.4, -0.14)	0.45
Dapagliflozin 5mg/d, Metformin 2000mg/d	-0.063 (-0.87, 0.78)	-0.58 (-1.4, 0.28)	-0.31 (-0.91, 0.26)	0.39
Dapagliflozin 5mg/d, Placebo	-0.47 (-1.3, 0.31)	-0.15 (-1.3, 1.0)	-0.36 (-0.99, 0.27)	0.62
Empagliflozin 10mg/d, Metformin 2000mg/d	0.32 (-0.56, 1.2)	0.016 (-0.92, 0.91)	0.18 (-0.41, 0.78)	0.63
Empagliflozin 10mg/d, Placebo	0.074 (-0.50, 0.64)	0.33 (-0.68, 1.4)	0.13 (-0.34, 0.60)	0.67
Empagliflozin 10mg/d, Sitagliptin 100mg/d	-0.049 (-0.83, 0.72)	0.031 (-1.1, 1.1)	-0.018 (-0.62, 0.57)	0.91
Empagliflozin 25mg/d, Metformin 2000mg/d	0.20 (-0.66, 1.1)	-0.034 (-0.97, 0.87)	0.12 (-0.49, 0.71)	0.71
Empagliflozin 25mg/d, Placebo	0.034 (-0.56, 0.61)	0.17 (-0.88, 1.3)	0.072 (-0.41, 0.54)	0.81
Empagliflozin 25mg/d, Sitagliptin 100mg/d	0.0014 (-0.78, 0.79)	-0.14 (-1.3, 0.97)	-0.079 (-0.67, 0.50)	0.82
Placebo, Sitagliptin 100mg/d	-0.22 (-0.98, 0.54)	-0.11 (-1.1, 0.82)	-0.15 (-0.69, 0.40)	0.86

Table S7C Node-splitting analysis of the hypoglycemia outcome

Name	Direct Effect	Indirect Effect	Overall	P-Value
Canagliflozin 100mg/d, Metformin 2000mg/d	0.42 (-1.70, 2.58)	-0.03 (-2.38, 2.35)	0.14 (-1.30, 1.61)	0.77
Canagliflozin 100mg/d, Placebo	-1.26 (-2.43, -0.16)	-0.63 (-2.88, 1.62)	-1.14 (-2.12, -0.17)	0.60
Canagliflozin 100mg/d, Sitagliptin 100mg/d	-0.34 (-2.49, 1.78)	-0.99 (-3.77, 1.58)	-0.50 (-2.08, 1.03)	0.69
Canagliflozin 300mg/d, Metformin 2000mg/d	0.28 (-1.85, 2.39)	0.16 (-2.42, 2.82)	0.35 (-1.25, 1.95)	0.94
Canagliflozin 300mg/d, Placebo	-25.94 (-89.60, -1.44)	-0.79 (-2.27, 0.77)	-0.94 (-2.39, 0.52)	**0.04**
Canagliflozin 300mg/d, Sitagliptin 100mg/d	-0.02 (-2.18, 2.13)	-0.82 (-3.87, 2.02)	-0.29 (-2.00, 1.36)	0.64
Empagliflozin 10mg/d, Metformin 2000mg/d	0.86 (-2.18, 4.01)	0.98 (-1.27, 3.20)	0.94 (-0.80, 2.75)	0.95
Empagliflozin 10mg/d, Placebo	-0.33 (-1.96, 1.20)	-0.72 (-3.20, 1.81)	-0.34 (-1.73, 1.08)	0.77
Empagliflozin 10mg/d, Sitagliptin 100mg/d	-0.09 (-2.93, 2.60)	0.65 (-1.90, 3.25)	0.31 (-1.55, 2.12)	0.67
Empagliflozin 25mg/d, Metformin 2000mg/d	0.77 (-2.21, 3.82)	0.81 (-1.46, 2.99)	0.76 (-1.02, 2.51)	0.98
Empagliflozin 25mg/d, Placebo	-0.37 (-2.01, 1.11)	-0.90 (-3.37, 1.61)	-0.52 (-1.93, 0.84)	0.71
Empagliflozin 25mg/d, Sitagliptin 100mg/d	-0.18 (-3.00, 2.45)	0.48 (-2.10, 3.01)	0.12 (-1.77, 1.91)	0.70
Dapagliflozin 10mg/d, Metformin 2000mg/d	1.30 (-1.20, 4.04)	1.60 (-0.70, 3.94)	1.45 (-0.17, 3.16)	0.86
Dapagliflozin 10mg/d, Placebo	0.22 (-1.22, 1.69)	-0.04 (-3.11, 3.14)	0.17 (-1.11, 1.48)	0.88
Placebo, Sitagliptin 100mg/d	0.11 (-2.77, 2.79)	0.93 (-1.28, 3.15)	0.64 (-0.99, 2.21)	0.63

P-values < 0.05 are in bold, indicating a significant inconsistency between the direct effect and indirect effects.

Table S8 Meta-regression on risk of adverse outcomes with duration of trial.

Outcome	Genital infections					Unitary tract infections				
	Coef.	Std. Err.	*p* >|t|	tau^2	I^2	Coef.	Std. Err.	*p* >|t|	tau^2	I^2
Intervention arms vs. Control arms*	0.0025	0.0020	0.219	0.20	15.36%	0.0003	0.0005	0.516	0.00	0.00%
Intervention arms vs. Placebo	-0.0002	0.0015	0.902	0.00	0.00%	0.0003	0.0006	0.686	0.00	0.00%
**Outcome**	**Hypoglycemia**					**Nasopharyngitis**				
	Coef.	Std. Err.	*p* >|t|	tau^2	I^2	Coef.	Std. Err.	*p* >|t|	tau^2	I^2
Intervention arms vs. Control arms*	0.0015	0.0026	0.571	0.00	0.00%	0.0045	0.0022	0.053	0.00	0.00%
Intervention arms vs. Placebo	0.0053	0.0062	0.405	0.00	0.00%	0.0049	0.0029	0.104	0.00	0.00%

Abbreviation: Coef., coefficient; Std. Err., standard error. * Intervention arms and control arms across all included trials.

Table S9 Results of traditional pairwise meta-analysis of subgroup analysis of gender under genitourinary infections safety outcomes

T1	T2	GI/female				GI/male			
			Pairwise OR (95% Cl)	Heterogeneity			Pairwise OR (95% Cl)	Heterogeneity	
		Studies	Fixed effects	I^2	P-value	Studies	Fixed effects	I^2	P-value
Empagliflozin 10mg/d	Empagliflozin 25mg/d	3	0.87 (0.47, 1.62)	0.00%	0.809	4	1.36 (0.66, 2.80)	0.00%	0.897
Canagliflozin 100mg/d	Placebo	3	**2.10 (1.02, 4.29)**	15.60%	0.306	3	**7.49 (2.63, 21.33)**	50.00%	0.135
Empagliflozin 10mg/d	Placebo	2	**10.24 (2.23, 47.08)**	0.00%	0.537	3	**4.12 (1.17, 14.48)**	0.00%	0.497
Empagliflozin 25mg/d	Placebo	2	**13.27 (2.88, 61.26)**	0.00%	0.469	2	3.23 (0.89, 11.74)	0.00%	0.325
Canagliflozin 100mg/d	Canagliflozin 300mg/d	2	0.98 (0.47, 2.05)	11.90%	0.287	2	0.59 (0.23, 1.52)	0.00%	0.729
Dapagliflozin 10mg/d	Dapagliflozin 5mg/d	1	2.42 (0.67, 8.77)	NA	NA	1	0.91 (0.12, 6.85)	NA	NA
Empagliflozin 25mg/d	Sitagliptin 100mg/d	1	**11.91 (1.49, 95.42)**	NA	NA	1	3.97 (0.44, 35.98)	NA	NA
Empagliflozin 10mg/d	Sitagliptin 100mg/d	1	**9.99 (1.24, 80.74)**	NA	NA	1	4.06 (0.45, 36.77)	NA	NA
Canagliflozin 300mg/d	Sitagliptin 100mg/d	1	2.02 (0.67, 6.13)	NA	NA	1	**18.46 (1.05, 324.93)**	NA	NA
Canagliflozin 100mg/d	Sitagliptin 100mg/d	1	2.55 (0.88, 7.42)	NA	NA	1	12.73 (0.69, 233.88)	NA	NA
Ertugliflozin 5mg/d	Placebo	1	3.29 (0.99, 10.90)	NA	NA	1	2.83 (0.29, 27.72)	NA	NA
Ertugliflozin 15mg/d	Placebo	1	**4.89 (1.51, 15.76)**	NA	NA	1	4.76 (0.54, 41.67)	NA	NA
Dapagliflozin 5mg/d	Placebo	1	5.93 (0.63, 55.79)	NA	NA	1	5.34 (0.25, 115.89)	NA	NA
Dapagliflozin 10mg/d	Placebo	1	**14.33 (1.72, 119.57)**	NA	NA	1	4.85 (0.22, 104.99)	NA	NA
Empagliflozin 25mg/d	Metformin 2000mg/d	1	0.91 (0.25, 3.28)	NA	NA	1	8.12 (0.41, 159.47)	NA	NA
Empagliflozin 10mg/d	Metformin 2000mg/d	1	1.06 (0.29, 3.83)	NA	NA	1	13.00 (0.72, 234.04)	NA	NA
Dapagliflozin 5mg/d	Metformin 2000mg/d	1	**3.38 (1.07, 10.73)**	NA	NA	1	3.13 (0.13, 77.85)	NA	NA
Dapagliflozin 10mg/d	Metformin 2000mg/d	1	**8.13 (2.35, 28.12)**	NA	NA	1	3.39 (0.69, 16.75)	NA	NA
Canagliflozin 300mg/d	Metformin 2000mg/d	1	12.32 (0.67, 225.35)	NA	NA	1	10.63 (0.58, 194.49)	NA	NA
Canagliflozin 100mg/d	Metformin 2000mg/d	1	6.57 (0.34, 128.46)	NA	NA	1	5.63 (0.27, 118.59)	NA	NA
Empagliflozin 25mg/d	Metformin 1000mg/d	1	1.27 (0.33, 4.89)	NA	NA	1	7.60 (0.39, 149.42)	NA	NA
Empagliflozin 10mg/d	Metformin 1000mg/d	1	1.47 (0.38, 5.71)	NA	NA	1	12.17 (0.68, 219.28)	NA	NA
Ertugliflozin 15mg/d	Ertugliflozin 5mg/d	1	1.48 (0.62, 3.58)	NA	NA	1	1.69 (0.39, 7.28)	NA	NA
Placebo	Sitagliptin 100mg/d	1	1.56 (0.14, 17.48)	NA	NA	1	2.31 (0.21, 25.84)	NA	NA
Metformin 1000mg/d	Metformin 2000mg/d	1	0.72 (0.19, 2.78)	NA	NA	NR	NR	NR	NR

T1	T2	UTI/female				UTI/male			
			Pairwise OR (95% Cl)	Heterogeneity			Pairwise OR (95% Cl)	Heterogeneity	
		Studies	Fixed effects	I²	P-value	Studies	Fixed effects	I²	P-value
Empagliflozin 10mg/d	Empagliflozin 25mg/d	4	1.09 (0.67, 1.79)	0.00%	0.805	4	0.68 (0.27, 1.71)	0.00%	0.516
Empagliflozin 25mg/d	Placebo	3	1.01 (0.57, 1.80)	0.00%	0.694	3	1.24 (0.43, 3.60)	11.5%	0.323
Empagliflozin 10mg/d	Placebo	2	1.07 (0.61, 1.90)	0.00%	0.950	3	0.91 (0.29, 2.86)	0.00%	0.991
Dapagliflozin 10mg/d	Placebo	1	1.71 (0.36, 8.18)	NA	NA	1	4.85 (0.22, 104.99)	NA	NA
Dapagliflozin 5mg/d	Placebo	1	3.04 (0.70, 13.19)	NA	NA	1	5.34 (0.25, 115.89)	NA	NA
Dapagliflozin 10mg/d	Dapagliflozin 5mg/d	1	0.56 (0.14, 2.20)	NA	NA	1	0.91 (0.12, 6.85)	NA	NA
Empagliflozin 10mg/d	Metformin 1000mg/d	1	0.97 (0.41, 2.33)	NA	NA	1	1.78 (0.16, 19.92)	NA	NA
Empagliflozin 10mg/d	Metformin 2000mg/d	1	0.73 (0.31, 1.72)	NA	NA	1	0.94 (0.13, 6.80)	NA	NA
Empagliflozin 25mg/d	Metformin 1000mg/d	1	0.82 (0.35, 1.96)	NA	NA	1	3.22 (0.33, 31.61)	NA	NA
Empagliflozin 25mg/d	Metformin 2000mg/d	1	0.62 (0.27, 1.45)	NA	NA	1	1.70 (0.28, 10.45)	NA	NA
Metformin 1000mg/d	Metformin 2000mg/d	1	0.76 (0.33, 1.71)	NA	NA	1	0.53 (0.05, 5.94)	NA	NA
Dapagliflozin 5mg/d	Metformin 2000mg/d	1	0.95 (0.41, 2.22)	NA	NA	1	1.39 (0.30, 6.41)	NA	NA
Dapagliflozin 10mg/d	Metformin 2000mg/d	1	**3.28 (1.25, 8.61)**	NA	NA	1	**1.90 (0.46, 7.81)**	NA	NA
Empagliflozin 10mg/d	Sitagliptin 100mg/d	1	1.27 (0.58, 2.78)	NA	NA	1	0.65 (0.18, 2.36)	NA	NA
Empagliflozin 25mg/d	Sitagliptin 100mg/d	1	1.25 (0.57, 2.78)	NA	NA	1	0.64 (0.18, 2.31)	NA	NA
Placebo	Sitagliptin 100mg/d	1	1.20 (0.57, 2.53)	NA	NA	1	0.76 (0.21, 2.74)	NA	NA

Abbreviation: OR: odds ratio; Cl: confidence interval; NA: not applicable; NR: not reported; GI: genital infections; UTI: unitary tract infections.

Table S10A Results of Bayesian network meta-analysis of the genital infection outcome in different gender

Male

Canagliflozin100mg/d											
0.51<br>(0.08, 2.13)	Canagliflozin300mg/d										
1.79<br>(0.14, 14.18)	3.31<br>(0.24, 49.62)	Empagliflozin10mg/d									
2.59<br>(0.24, 24.03)	4.78<br>(0.38, 85.90)	1.42<br>(0.49, 6.24)	Empagliflozin25mg/d								
2.23<br>(0.03, 46.41)	4.49<br>(0.05, 138.56)	1.30<br>(0.02, 31.18)	0.89<br>(0.01, 19.15)	Dapagliflozin5mg/d							
4.79<br>(0.12, 105.07)	9.98<br>(0.22, 340.97)	2.74<br>(0.08, 74.99)	1.85<br>(0.05, 47.25)	2.07<br>(0.18, 54.05)	Dapagliflozin10mg/d						
2.26<br>(0.03, 75.70)	4.60<br>(0.05, 232.76)	1.44<br>(0.02, 51.89)	0.96<br>(0.01, 31.68)	1.13<br>(0.01, 273.74)	0.50<br>(0.00, 68.01)	Ertugliflozin5mg/d					
1.48<br>(0.02, 35.70)	2.61<br>(0.05, 118.53)	0.90<br>(0.01, 23.61)	0.60<br>(0.01, 14.05)	0.63<br>(0.00, 95.75)	0.28<br>(0.00, 25.08)	0.58<br>(0.04, 5.91)	Ertugliflozin15mg/d				
26.60<br>(2.07, 1188.78)	**54.13<br>(3.70, 3006.66)**	**14.84<br>(1.44, 595.79)**	9.89<br>(0.93, 354.57)	12.11<br>(0.29, 2755.11)	6.14<br>(0.14, 680.53)	10.77<br>(0.22, 2060.87)	20.18<br>(0.50, 2577.73)	Sitagliptin100mg/d			
——	——	——	——	——	——	——	——	——	Metformin1000mg/d		
**41.44<br>(2.57, 1654.14)**	**87.30<br>(4.58, 5368.76)**	**25.23<br>(1.72, 1188.91)**	**16.80<br>(1.18, 682.60)**	**23.47<br>(1.44, 1536.06)**	**9.72<br>(1.34, 211.62)**	19.58<br>(0.27, 4303.48)	35.26<br>(0.71, 5278.48)	1.81<br>(0.03, 88.94)	**0.00<br>(0.00, 0.92)**	Metformin2000mg/d	
**9.18<br>(1.77, 53.68)**	**18.84<br>(2.38, 224.88)**	**5.38<br>(1.24, 38.56)**	3.63<br>(0.75, 23.15)	4.32<br>(0.27, 277.00)	2.05<br>(0.11, 70.35)	4.00<br>(0.19, 218.40)	6.45<br>(0.44, 337.11)	0.36<br>(0.01, 4.47)	**0.00<br>(0.00, 0.18)**	0.22<br>(0.01, 3.47)	Placebo

Female

Canagliflozin100mg/d											
0.77<br>(0.13, 2.81)	Canagliflozin300mg/d										
0.49<br>(0.07, 2.93)	0.63<br>(0.08, 6.10)	Empagliflozin10mg/d									
0.43<br>(0.06, 2.53)	0.55<br>(0.07, 5.21)	0.86<br>(0.25, 3.12)	Empagliflozin25mg/d								

0.37 (0.04, 4.00)	0.49 (0.06, 8.86)	0.76 (0.09, 9.22)	0.86 (0.11, 11.18)	Dapagliflozin5mg/d							
0.15 (0.02, 1.64)	0.19 (0.02, 3.36)	0.31 (0.03, 3.67)	0.36 (0.04, 4.33)	0.41 (0.06, 2.54)	Dapagliflozin10mg/d						
0.89 (0.07, 13.83)	1.14 (0.08, 29.91)	1.77 (0.14, 39.00)	2.03 (0.15, 42.72)	2.34 (0.10, 51.22)	5.61 (0.26, 137.28)	Ertugliflozin5mg/d					
0.59 (0.04, 9.04)	0.75 (0.06, 20.97)	1.18 (0.09, 26.85)	1.36 (0.10, 30.09)	1.56 (0.07, 32.75)	3.77 (0.18, 88.36)	0.67 (0.08, 5.56)	Ertugliflozin15mg/d				
2.78 (0.51, 16.86)	3.59 (0.72, 32.54)	5.55 (0.99, 47.90)	**6.41 (1.11, 55.36)**	7.54 (0.57, 82.37)	**18.92 (1.42, 220.31)**	3.24 (0.16, 54.82)	4.80 (0.25, 83.91)	Sitagliptin100mg/d			
0.85 (0.07, 11.50)	1.13 (0.09, 22.79)	1.74 (0.26, 16.20)	2.02 (0.28, 19.61)	2.34 (0.14, 30.64)	5.81 (0.35, 78.66)	0.97 (0.04, 22.32)	1.44 (0.05, 36.04)	0.31 (0.02, 4.14)	Metformin1000mg/d		
1.32 (0.23, 10.16)	1.70 (0.32, 22.53)	2.74 (0.59, 21.26)	3.17 (0.66, 24.22)	3.56 (0.69, 19.89)	**8.62 (1.70, 53.06)**	1.55 (0.09, 27.39)	2.31 (0.14, 40.22)	0.48 (0.07, 4.50)	1.51 (0.18, 16.23)	Metformin2000mg/d	
3.04 (0.88, 14.54)	3.94 (0.85, 40.01)	**6.21 (1.55, 44.90)**	**7.14 (1.76, 52.63)**	**8.30 (1.04, 70.11)**	**20.18 (2.77, 184.78)**	3.47 (0.39, 35.54)	5.28 (0.58, 52.23)	1.10 (0.19, 7.46)	3.54 (0.37, 42.56)	2.30 (0.41, 13.53)	Placebo

Effect sizes represent summary odds ratio and 95% credible intervals. “-” indicate the effect size with very width credible interval owing to the small sample sizes, which were not shown. OR>1 indicate that the treatment specified in the column got more risk effect than that specified in the row, which values with significant differences are in bold.

Table S10B Results of Bayesian network meta-analysis of the urinary tract infection outcome in different gender

**Male**							
Empagliflozin 10mg/d							
0.66 (0.19, 2.36)	Empagliflozin 25mg/d						
0.30 (0.02, 3.46)	0.47 (0.03, 4.61)	Dapagliflozin 5mg/d					
0.26 (0.02, 3.13)	0.40 (0.03, 4.43)	0.89 (0.11, 7.25)	Dapagliflozin 10 mg/d				
2.28 (0.15, 90.60)	3.20 (0.25, 133.15)	7.84 (0.30, 405.34)	8.62 (0.39, 479.66)	Metformin 1000 mg/d			
0.63 (0.08, 4.85)	0.99 (0.12, 6.06)	2.08 (0.36, 14.57)	**2.36 (0.41, 15.14)**	0.29 (0.01, 4.10)	Metformin 2000 mg/d		
0.57 (0.10, 3.84)	0.87 (0.15, 5.49)	1.88 (0.14, 45.45)	**2.19 (0.15, 47.40)**	0.26 (0.01, 5.53)	0.87 (0.09, 13.01)	Sitagliptin 100 mg/d	
1.01 (0.26, 4.83)	1.58 (0.40, 6.85)	3.34 (0.32, 62.11)	**3.81 (0.37, 63.51)**	0.44 (0.01, 8.18)	1.61 (0.21, 16.41)	1.80 (0.29, 12.21)	Placebo
**Female**							
Empagliflozin 10 mg/d							
1.08 (0.50, 2.20)	Empagliflozin 25 mg/d						
0.50 (0.12, 1.81)	0.47 (0.12, 1.75)	Dapagliflozin 5 mg/d					
0.36 (0.10, 1.35)	0.33 (0.09, 1.31)	0.73 (0.22, 2.69)	Dapagliflozin 10 mg/d				
0.91 (0.29, 3.01)	0.87 (0.29, 2.83)	1.91 (0.44, 9.22)	**2.60 (0.57, 11.60)**	Metformin 1000 mg/d			
0.73 (0.25, 1.91)	0.67 (0.24, 1.86)	1.44 (0.53, 4.39)	**2.00 (0.68, 5.65)**	0.79 (0.23, 2.54)	Metformin 2000 mg/d		
1.34 (0.42, 3.93)	1.23 (0.42, 3.80)	2.65 (0.57, 14.18)	**3.77 (0.72, 17.98)**	1.46 (0.31, 6.37)	1.88 (0.47, 7.64)	Sitagliptin 100 mg/d	
1.07 (0.49, 2.40)	0.99 (0.48, 2.34)	2.12 (0.63, 9.05)	**2.90 (0.84, 10.82)**	1.14 (0.33, 4.29)	1.46 (0.53, 4.57)	0.81 (0.27, 2.56)	Placebo

Effect sizes represent summary odds ratio and 95% credible intervals. OR>1 indicate that the treatment specified in the column got more risk effect than that specified in the row, which values with significant differences are in bold.

Table S11 SUCRA values of subgroup analysis of gender under genitourinary infections safety outcomes

Treatment	SUCRA value			
	**UTI-female**	**UTI-male**	**GI-female**	**GI-male**
Empagliflozin10mg/d	39.5	34.5	66.5	67.9
Empagliflozin25mg/d	32.8	53.1	71.5	57.2
Dapagliflozin5mg/d	78.5	75.7	71.3	65.6
Dapagliflozin10mg/d	**90.8**	**79.4**	**91.9**	50.5
Metformin1000mg/d	43.2	18.9	45.5	0.7
Metformin2000mg/d	60.1	50.0	30.9	16.1
Sitagliptin100mg/d	22.1	57.2	12.3	20.4
Canagliflozin100mg/d	NA	NA	41.5	75.4
Canagliflozin300mg/d	NA	NA	52.2	**88.8**
Ertugliflozin5mg/d	NA	NA	46.9	57.4
Ertugliflozin15mg/d	NA	NA	60.3	69.6
Placebo	33.0	31.2	9.1	30.5

Note: SUCRA: surface under the cumulative ranking curves; UTI: urinary tract infection, GI: genital infection, NA: not applicable.

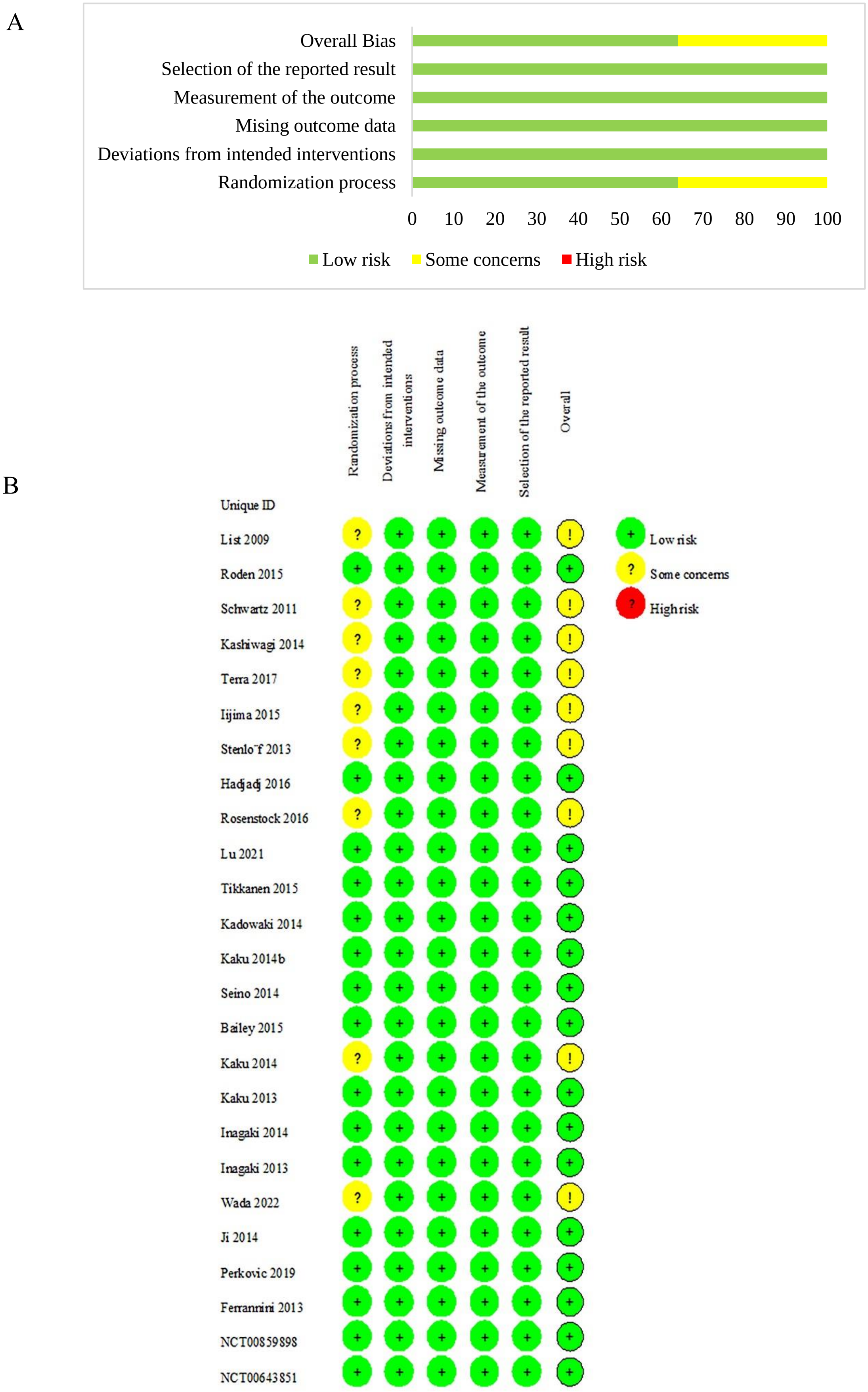

Figure S1 Summary risk of bias of the included studies. (A) Risk of bias graph. (B) Risk of bias summary

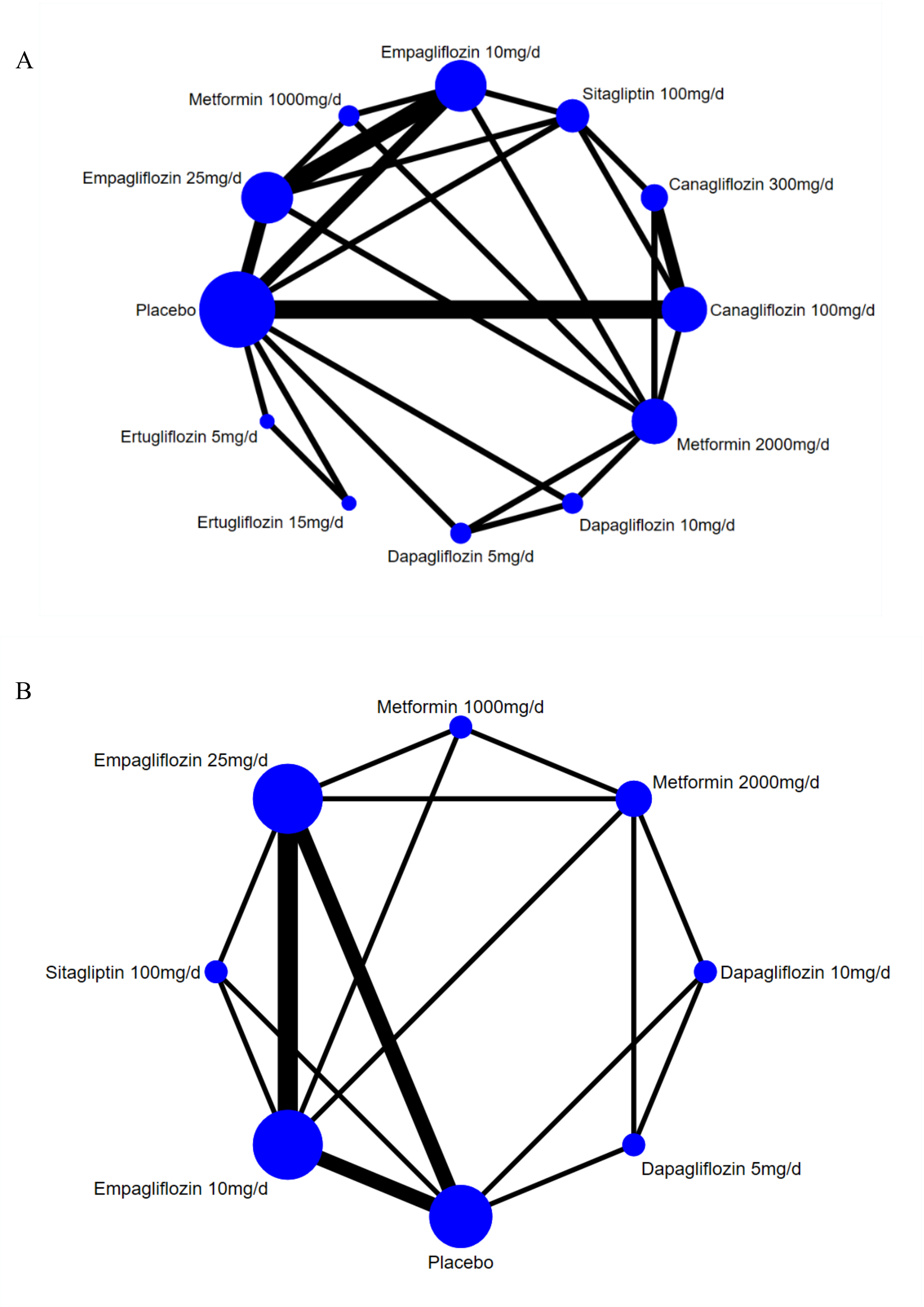

Figure S2 Network diagrams of comparisons on subgroup analysis of gender under genitourinary infections safety outcomes. (A). urinary tract infections. (B) genital infections

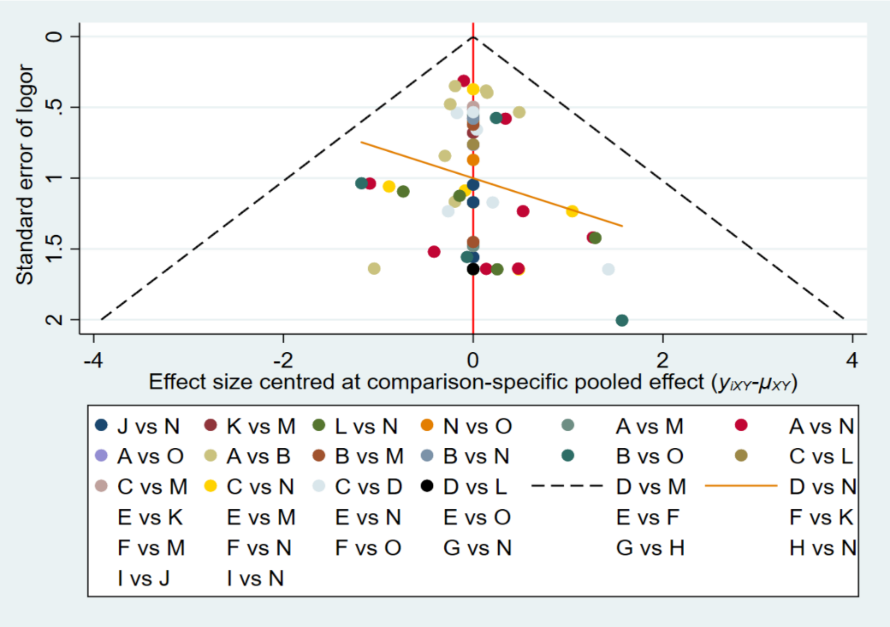

Figure S3A Comparison-adjusted funnel plot for genital infections outcome (A: Canagliflozin 100mg/d; B: Canagliflozin 300mg/d; C: Dapagliflozin 10mg/d; D: Dapagliflozin 5mg/d; E: Empagliflozin 10mg/d; F: Empagliflozin 25mg/d; G: Ertugliflozin 15mg/d; H: Ertugliflozin 5mg/d; I: Ipragliflozin 100mg/d; J: Ipragliflozin 50mg/d; K: Metformin 1000mg/d; L: Metformin 1500mg/d; M: Metformin 2000mg/d; N: Placebo; O: Sitagliptin 100mg/d)

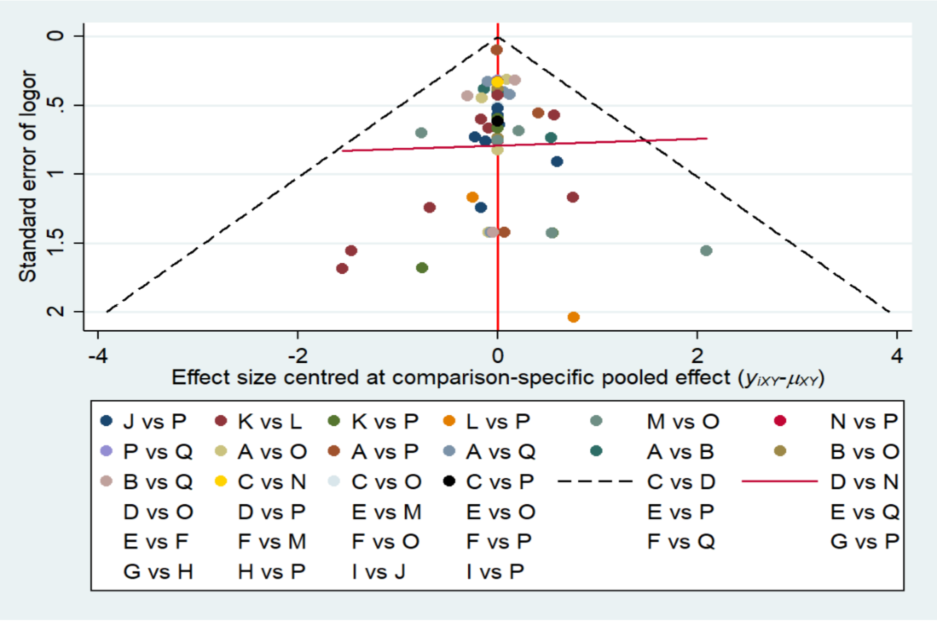

Figure S3B Comparison-adjusted funnel plot for urinary tract infections outcome (A: Canagliflozin 100mg/d; B: Canagliflozin 300mg/d; C: Dapagliflozin 10mg/d; D: Dapagliflozin 5mg/d; E: Empagliflozin 10mg/d; F: Empagliflozin 25mg/d; G: Ertugliflozin 15mg/d; H: Ertugliflozin 5mg/d; I: Henagliflozin 10mg/d; J: Henagliflozin 5mg/d; K: Ipragliflozin 100mg/d; L: Ipragliflozin 50mg/d; M: Metformin 1000mg/d; N: Metformin 1500mg/d; O: Metformin 2000mg/d; P: Placebo; Q: Sitagliptin 100mg/d)

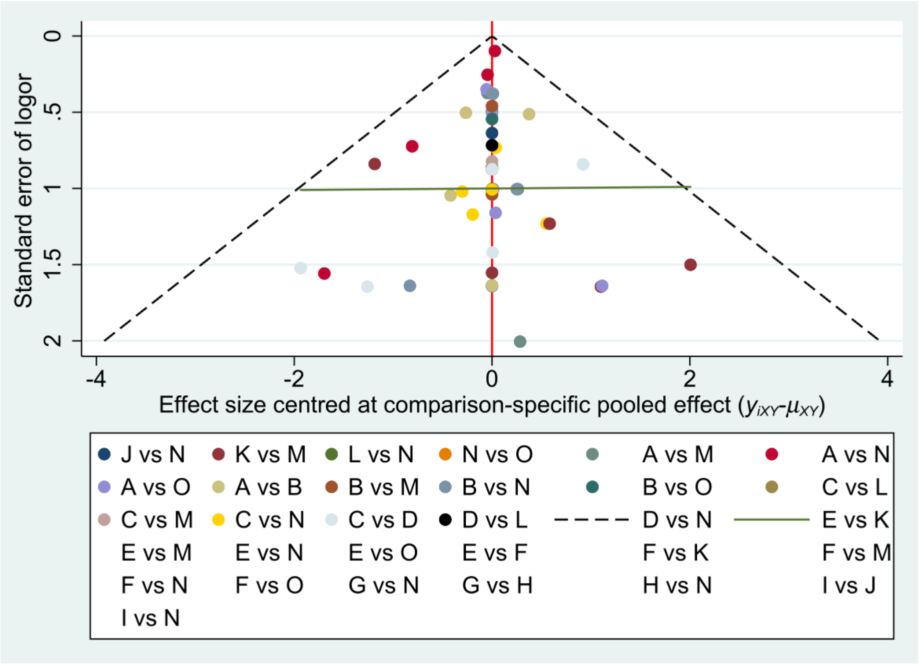

Figure S3C Comparison-adjusted funnel plot for hypoglycemia outcome (A: Canagliflozin 100mg/d; B: Canagliflozin 300mg/d; C: Dapagliflozin 10mg/d; D: Dapagliflozin 5mg/d; E: Empagliflozin 10mg/d; F: Empagliflozin 25mg/d; G: Ertugliflozin 15mg/d; H: Ertugliflozin 5mg/d; I: Henagliflozin 10mg/d; J: Henagliflozin 5mg/d; K: Metformin 1000mg/d; L: Metformin 1500mg/d; M: Metformin 2000mg/d; N: Placebo; O: Sitagliptin 100mg/d)

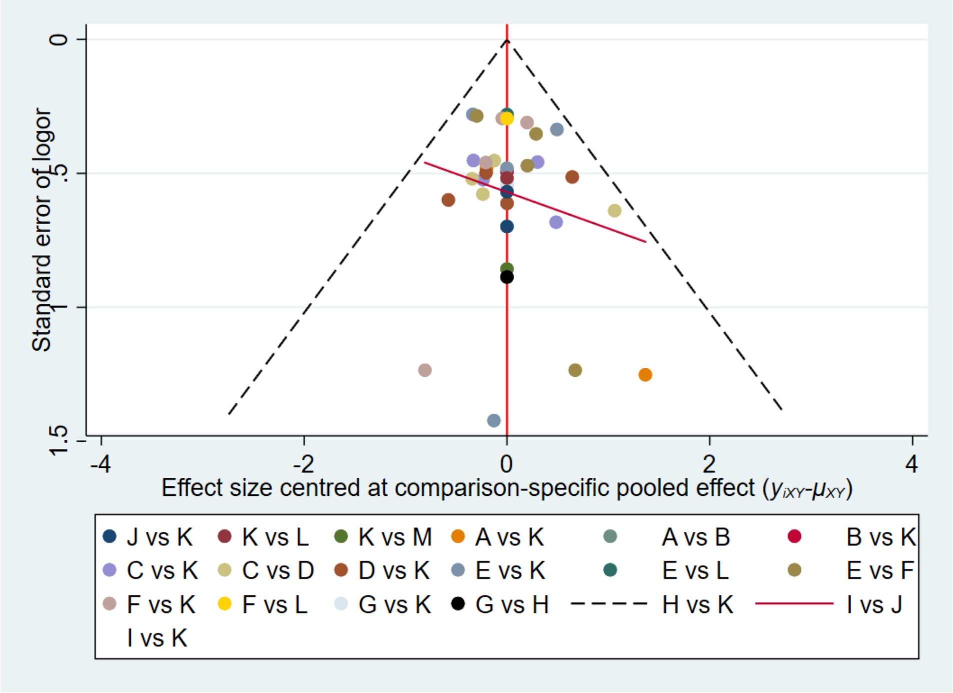

Figure S3D Comparison-adjusted funnel plot for nasopharyngitis outcome (A: Canagliflozin 100mg/d; B: Canagliflozin 300mg/d; C: Dapagliflozin 10mg/d; D: Dapagliflozin 5mg/d; E: Empagliflozin 10mg/d; F: Empagliflozin 25mg/d; G: Henagliflozin 10mg/d; H: Henagliflozin 5mg/d; I: Luseogliflozin 2.5mg/d; J: Luseogliflozin 5mg/d; K: Placebo; L: Sitagliptin 100mg/d; M: Tofogliflozin 20mg/d)
